# Machine Learning Prediction of Incident Hypertension in Australian Men: Integrating Survey, Pharmaceutical and Healthcare Utilisation Data From the Ten to Men Cohort

**DOI:** 10.1155/ijhy/3041345

**Published:** 2026-08-25

**Authors:** Ebenezer Afrifa-Yamoah, Emmanuel Peprah-Yamoah, Victor Opoku-Yamoah, Eric Adua

**Affiliations:** ^1^ Mathematical Application and Data Analytics Group, School of Science, Edith Cowan University, Perth, Western Australia, Australia, ecu.edu.au; ^2^ Department of Chemistry, University of Connecticut, Mansfield, Connecticut, USA, uconn.edu; ^3^ Kinesiology and Health Sciences, University of Waterloo, Waterloo, Canada, uwaterloo.ca; ^4^ School of Optometry and Vision Science, University of Waterloo, Waterloo, Canada, uwaterloo.ca; ^5^ College of Medicine and Public Health, Flinders University, Adelaide, South Australia, Australia, flinders.edu.au

**Keywords:** administrative data linkage, hypertension, longitudinal cohort, Pharmaceutical Benefits Scheme, pharmaceutical dispensing, risk prediction, Ten to Men

## Abstract

**Background:**

Hypertension is a leading modifiable contributor to cardiovascular disease. We developed and evaluated machine learning models to predict incident hypertension in Australian men using baseline survey data linked to pharmaceutical (PBS) and healthcare utilisation (MBS) administrative records from the Ten to Men cohort.

**Methods:**

Among 13,519 men free of hypertension at baseline (2014), incident hypertension over 10 years was ascertained from PBS antihypertensive dispensing. Twenty‐five baseline features spanning sociodemographic, anthropometric, lifestyle, clinical and administrative domains were used to train logistic regression, random forest, gradient boosting and XGBoost models, evaluated by five‐fold cross‐validation with out‐of‐fold prediction. Discrimination, calibration and threshold‐based operating characteristics were reported with 95% confidence intervals.

**Results:**

Incident hypertension occurred in 1310 men (9.7%). All algorithms achieved comparable discrimination (AUROC ≈ 0.77; XGBoost 0.771, 95% CI: 0.756–0.784), with no material advantage for flexible tree‐based methods over regularised logistic regression. Age, administrative markers of healthcare engagement and BMI were the leading predictors, and a parsimonious six‐variable model recovered almost all the discrimination of the full model. The model showed high negative predictive and low positive predictive values across decision thresholds, consistent with the 9.7% event rate. Incorporating measured blood pressure history from intervening waves increased apparent discrimination, but this gain was attributable to information contemporaneous with the outcome and is reported only as a concurrent surveillance benchmark.

**Conclusions:**

Routinely collected survey and administrative data can identify Australian men at low risk of incident hypertension with high negative predictive value, supporting population‐level risk stratification rather than individual diagnosis. Predictive performance was driven by feature availability and quality rather than algorithm choice.

## 1. Introduction

Globally, hypertension remains a significant public health threat, accelerating the death of people and increasing healthcare costs from hospitalisation and medications, as well as reducing the quality of life of those affected. Usually recognised as a silent killer, it is responsible for up to 10.8 million deaths annually, while some 1.28 billion adults are currently diagnosed with it [1, 2]. Although highly treatable, its detection and control remain poor globally [3]. In Australia, approximately 31% of adults have hypertension, yet awareness, treatment and control rates are only 56%, 54% and 34%, respectively [4]. These outcomes lag comparable high‐income countries such as Canada, where control rates approach 68% [5]. The economic burden is substantial, with hypertension‐attributable productivity losses estimated at AUD $137 billion over the working lifetime of the current population [6]. A national roadmap released in 2024 set a target of 70% blood pressure control by 2030, identifying improved detection as a critical priority [5].

Early identification of individuals at high risk of developing hypertension is central to primary prevention [3, 7]. Existing prediction models, such as the Framingham Hypertension Risk Score, demonstrate moderate discrimination, with pooled AUCs around 0.78 across external validations [9–11]. A recent systematic review reported pooled AUCs of 0.78 for traditional models and 0.82 for machine learning models, but with extreme heterogeneity and few studies at low risk of bias [10]. Critically, most models rely on clinical measurements, particularly systolic blood pressure, which is the dominant predictor of incident hypertension [11, 12]. These data are typically unavailable in population‐based screening settings, creating a paradox in which those most in need of early detection lack the inputs required by existing tools [13].

Linked administrative health data provide an alternative pathway to population‐level risk prediction. In Australia, the Pharmaceutical Benefits Scheme (PBS) and Medicare Benefits Schedule (MBS) offer near‐complete longitudinal coverage of medication use and healthcare utilisation. These datasets have been validated for identifying treated chronic conditions, including hypertension [14, 15]. Their value for predicting incident hypertension, particularly when combined with survey data and analysed using machine learning, has not been systematically examined [10]. Machine learning methods have been proposed to improve prediction by capturing nonlinear relationships and feature interactions [16–22]. The longitudinal cohorts further enable assessment of whether within‐person trajectories improve prediction beyond baseline measures. The Australian Longitudinal Study on Male Health (Ten to Men) uniquely integrates repeated survey data with linked PBS and MBS records across five waves over approximately 10 years [23, 24], addressing gaps in both longitudinal prediction and sex‐specific modelling [25].

This study aimed to (1) develop and internally validate machine learning models for 10‐year incident hypertension using combined survey, PBS and MBS data; (2) compare machine learning performance with logistic regression; (3) quantify the incremental predictive value of administrative health data; (4) evaluate whether longitudinal trajectory features improve prediction beyond baseline assessment; and (5) identify key predictors and their nonlinear effects using SHapley Additive exPlanations (SHAP)–based interpretability methods [26]. Through sensitivity analyses, we also assess the limits of prediction achievable without clinical measurements, informing the role of nonclinical data in population screening strategies.

## 2. Methods

### 2.1. Study Design and Reporting

This prognostic prediction study used data from the Ten to Men Australian Longitudinal Study on Male Health, a national cohort of males aged 10–55 years at recruitment [23, 24, 27]. Established in 2013–2014 (Wave 1) using stratified, multistage cluster sampling with oversampling in rural and regional areas, the study is geographically representative across major cities and regional/remote Australia [28]. Data collection combined self‐administered questionnaires (ages 15–55 years) and computer‐assisted interviews (ages 10–14 years) [23], with follow‐up across five waves to 2023–2024, providing up to 10 years of longitudinal data.

Survey responses were linked to national administrative data from the PBS and MBS from March 2011 to February 2025. PBS data capture subsidised prescription medicines, including below‐copayment items from April 2012 [14, 15], while MBS records medical services, diagnostics and pathology tests within Australia’s universal healthcare system. Participants provided informed consent at each wave, including for data linkage. This secondary analysis used de‐identified linked data and was reported in accordance with TRIPOD guidelines [29].

### 2.2. Study Population

The analytic cohort was drawn from the Ten to Men cohort. Of 16,021 men assessed at the 2014 wave, 2502 with prevalent hypertension at baseline were excluded: 2301 who reported a prior hypertension diagnosis and 978 identified from antihypertensive dispensing records before baseline, of whom 777 met both criteria. This left an analytic cohort of 13,519 men free of hypertension at baseline.

### 2.3. Outcome Definition

Incident hypertension was defined as the first dispensing of an antihypertensive agent recorded in PBS data within 10 years of baseline. A 5‐year definition (550 events, 4.1%) was used in sensitivity analysis. PBS‐based outcome definition provides several advantages: (a) objective event ascertainment independent of survey participation, eliminating the detection bias whereby only participants who return for follow‐up waves can report new diagnoses; (b) precise event dates from dispensing records, enabling accurate time‐to‐event calculations; and (c) reduced classification noise from the bidirectional misclassification inherent in self‐reported hypertension, where participants with undiagnosed hypertension at baseline contaminate the at‐risk cohort and those who develop hypertension but do not report it create false negatives [13].

A total of 1310 men (9.7%) experienced the primary outcome, yielding a class imbalance ratio of approximately 1:9.3. The secondary prediction target was 5‐year incident hypertension (550 events; 4.1%), providing a shorter‐horizon assessment of model generalisability across time windows. The maximum follow‐up was 11.1 years (censored at 15 February 2025), yielding 142,956 person‐years of observation. The incidence rate was 9.2 per 1000 person‐years for the 10‐year outcome.

### 2.4. Predictor Variables

Twenty‐five baseline features were derived (Table A1 of Supporting information). Categorical survey items were encoded as indicator variables; ordinal items (household income and alcohol use) were coded on their natural ordering; and administrative counts (diabetes‐related dispensing, GP management plan and team care arrangement claims and HbA1c tests) were used as recorded, with the absence of a claim coded as zero.

For the subset of men with at least three waves of data (*n* = 7188), longitudinal features were derived: within‐person change and variability in BMI, consistency and change in physical activity, the number of observed waves and a blood pressure trajectory class summarising self‐reported high blood pressure status across waves. Because the blood pressure trajectory class incorporates status measured during the outcome ascertainment window, it is contemporaneous with rather than antecedent to the outcome; models including it are reported only as a concurrent surveillance benchmark and are not comparable with the baseline prediction models.

### 2.5. Machine Learning Models

Four classification models were evaluated to span the spectrum from traditional regression to modern ensemble methods. L2‐regularised logistic regression (*C* = 1.0) served as the benchmark, reflecting its continued dominance in clinical prediction and its role as a reference standard against which machine learning models must demonstrate meaningful improvements [30, 31]. A random forest (500 trees, maximum depth 8 and minimum 20 leaf samples) was included as a bagging‐based ensemble that reduces variance through bootstrap aggregation of decorrelated trees [32, 33], with depth constrained to limit overfitting to sparse event–feature combinations, consistent with prior experience in this cohort. XGBoost (up to 2000 estimators, maximum depth 3, learning rate 0.02, column and row subsampling 0.8, early stopping after 50 rounds and scale_pos_weight = 1.0) was selected as the primary gradient‐boosted model due to its strong performance in tabular data and its built‐in regularisation, missing data handling and early stopping; shallow trees were used to favour low‐order interactions and reduce overfitting. A standard gradient boosting model (500 estimators, maximum depth 3 and learning rate 0.02) provided a comparator without XGBoost‐specific regularisation.

All models applied median imputation and z‐score normalisation within each cross‐validation fold to prevent data leakage, while categorical variables retained a ‘missing’ category to preserve sample size and allow learning from missingness patterns. Class weighting was not used: setting XGBoost’s scale_pos_weight to the imbalance ratio (9.3:1) reduced AUC by 0.042, and prior analyses in the same cohort showed similar degradation in random forest performance (AUC 0.664–0.645) with inverse‐prevalence weighting, suggesting amplification of idiosyncratic event patterns. Accordingly, scale_pos_weight was fixed at 1.0. Early stopping was implemented using a 20% validation split within each training fold (log‐loss), selecting approximately 180 trees and preventing overfitting compared with training the full 2000 estimators. Together, these findings indicate that default class weighting may be unreliable at moderate event rates and that validation‐based early stopping is essential for gradient‐boosted models, even in clinical prediction settings.

### 2.6. Evaluation

Incident hypertension was uncommon (9.7% at 10 years). Rather than resampling, the natural class distribution was retained and performance evaluated across decision thresholds. Accuracy was interpreted against the no‐information rate (0.903), and calibration was assessed using the Brier score.

Models were evaluated using 5‐fold stratified cross‐validation, preserving outcome prevalence within folds. Out‐of‐fold predictions were pooled to generate a single set of probabilities for the full cohort, enabling ROC and calibration analyses without leakage. Discrimination was assessed using AUC‐ROC, which measures ranking performance independent of thresholds [34], while the Brier score quantified overall performance as the mean squared error between predicted probabilities and observed outcomes.

Average precision (area under the precision–recall curve) was included to better reflect performance under class imbalance. Calibration was examined using 10‐bin quantile‐based calibration plots comparing predicted and observed risks. Sensitivity at clinically relevant thresholds (0.05, 0.088 [Youden‐optimal], 0.10, 0.15, 0.20) was also reported to characterise screening‐relevant operating points [34, 35], with full threshold metrics provided in Appendix A (Table A3).

### 2.7. Sensitivity Analyses

Six prespecified sensitivity analyses were conducted to assess robustness to key analytical decisions and potential biases. Restricting the sample to adults (≥ 18 years) addressed potential differences in hypertension risk, BMI distributions and lifestyle predictors between adolescents and adults, given the baseline age range of 10–55 years. A complete‐case analysis excluded all observations with missing predictors—primarily those missing household income (29.0%)—to evaluate whether median imputation introduced bias. To quantify the incremental value of administrative data, PBS/MBS‐linked variables (insulin‐treated and non‐insulin diabetes, HbA1c testing, GPMP/TCA status and related counts) were removed, enabling comparison with survey‐only models relevant to settings without data linkage. Increasing cross‐validation from 5‐ to 10‐fold assessed the stability of AUC estimates under finer data partitioning, while rerunning analyses with an alternative random seed (123) evaluated sensitivity to random initialisation. In addition, XGBoost models were trained separately in participants with diabetes (*n* = 459; 47.3% event rate) and without diabetes (*n* = 13,405; 9.4% event rate) to examine differences in predictor importance and performance across metabolic strata, which is critical for targeted screening where administrative data may have differential utility.

A prespecified exploratory modelling study further assessed robustness to alternative outcome definitions, feature sets and algorithm classes, using a self‐reported hypertension outcome and a reduced predictor set. This analysis compared logistic regression with multiple random forest variants and a stacked ensemble. Full details of modelling procedures, tuning and evaluation are provided in Appendix B. The exploratory results are presented to support internal replication and to determine whether more complex machine‐learning approaches yield meaningful improvements in discrimination over conventional regression within the same cohort.

### 2.8. Longitudinal Surveillance Benchmark and Trajectory‐Enhanced Prediction

Among participants with data at three or more waves (*n* = 7188; 879 events; 12.2%), two XGBoost models were compared using identical hyperparameters and a cross‐validation framework: a baseline model using 25 features only and a trajectory‐enhanced model using 31 features (25 baseline plus 6 trajectory variables). The ΔAUC between models quantified the incremental value of longitudinal information. This comparison was conducted within the trajectory‐eligible subsample to ensure that performance differences reflect the added features rather than differences in sample composition. The higher event rate in this subsample (12.2% vs. 9.7% overall) reflects the longer minimum follow‐up required for inclusion (≥ 3 waves), which increases the cumulative probability of developing hypertension. Because the trajectory model includes a blood pressure trajectory class variable that incorporates self‐reported hypertension status from intermediate waves, the trajectory AUC should be interpreted as reflecting the combined value of longitudinal health monitoring rather than purely baseline prediction. It represents the discrimination achievable if repeated survey data and administrative records are both available, as would be the case in a longitudinal population health surveillance system.

### 2.9. Interpretability

Model interpretability was assessed using SHAP values computed with the TreeSHAP algorithm [26] on an XGBoost model trained on the full cohort (300 estimators, reflecting the early‐stopping optimum). TreeSHAP provides exact Shapley values for tree ensembles, decomposing each prediction into additive feature contributions relative to the population base rate. Global importance was quantified as the mean absolute SHAP value across all 13,519 participants, ranking features by average marginal contribution.

SHAP dependence plots were generated for all 25 baseline features (Appendix A, Figure A1), with the four most influential features shown in the main text. These plots characterise nonlinear relationships between feature values and their contributions to predicted risk, revealing threshold effects, dose–response patterns and interactions not evident from importance rankings alone. Directional analysis of SHAP contributions further indicated whether features predominantly increased or decreased risk across individuals, clarifying model behaviour. This interpretability framework addresses a key barrier to clinical ML adoption by showing both which features drive predictions and how they do so [18, 26].

### 2.10. Software

Primary analyses were conducted using Python 3.12 with scikit‐learn 1.6 (logistic regression, random forest, gradient boosting and cross‐validation infrastructure), XGBoost 2.1 (XGBoost implementation) and SHAP 0.46 (SHAP value computation). All random number generation was seeded for reproducibility (primary seed: 42; alternative seed: 123 for sensitivity analysis S5, with fold‐specific increments to ensure independent fold partitions).

## 3. Results

### 3.1. Cohort Characteristics

Of 13,519 men free of hypertension at baseline, 1310 (9.7%) developed incident hypertension within 10 years. Baseline characteristics by incident status are shown in Table 1. Men who developed hypertension were older, had a higher BMI and more frequently carried administrative markers of chronic disease management at baseline. Men without diabetes were youngest (mean age 33.0 years, SD 12.8) and leanest (mean BMI 25.8 kg/m^2^; 14.2% obese), whereas non‐insulin–treated men were older (mean 42.2 years, SD 9.5) with the highest BMI (31.1 kg/m^2^; 47.0% obese). University education was most common among men without diabetes (34.1%), compared with non‐insulin–treated (36.9%) and insulin‐treated (27.8%) groups, consistent with the inverse relationship between education and diabetes prevalence.

**TABLE 1:** Baseline characteristics of the analytic cohort, by 10‐year incident hypertension status.

Characteristic	Overall (*N* = 13,519)	No incident HTN (*n* = 12,209)	Incident HTN (*n* = 1310)
Age (years)—mean (SD)	33.3 (12.8)	32.5 (12.7)	40.4 (11.7)
BMI (kg/m^2^)—mean (SD)	26.0 (5.1)	25.7 (5.0)	28.3 (5.5)
BMI category: underweight	2217 (16.4)	2065 (16.9)	152 (11.6)
BMI category: normal	4622 (34.2)	4323 (35.4)	299 (22.8)
BMI category: overweight	4627 (34.2)	4137 (33.9)	490 (37.4)
BMI category: obese	2053 (15.2)	1684 (13.8)	369 (28.2)
University degree	2829 (20.9)	2526 (20.7)	303 (23.1)
Married/de facto	7411 (54.8)	6542 (53.6)	869 (66.3)
Met physical activity threshold	7867 (58.2)	7168 (58.7)	699 (53.4)
Current smoker	2256 (16.7)	1987 (16.3)	269 (20.5)
High cholesterol	1260 (9.3)	1044 (8.6)	216 (16.5)
Heart condition	72 (0.5)	62 (0.5)	10 (0.8)
Stroke	43 (0.3)	35 (0.3)	8 (0.6)
Diabetes: non‐insulin–treated	345 (2.6)	175 (1.4)	170 (13.0)
Diabetes: insulin‐treated	114 (0.8)	67 (0.5)	47 (3.6)
GP management plan/team care arrangement	2038 (15.1)	1460 (12.0)	578 (44.1)
HbA1c ever tested	522 (3.9)	313 (2.6)	209 (16.0)
Sleep problems reported^†^	6366 (47.1)	5650 (46.3)	716 (54.7)

*Note:* Values are mean (SD) for continuous variables and *n* (%) for categorical variables. Percentages for categorical rows are of the column total.

^†^Sleep problems were ascertainable for 6366 of 13,519 men (47%) after correction; the percentage shown is of the column total and is therefore a lower bound on prevalence among those assessed.

Administrative (PBS/MBS‐linked) features revealed distinct healthcare utilisation patterns. HbA1c testing was recorded at 3.9% overall but concentrated in diabetes groups (62.6% non‐insulin–treated; 90.4% insulin‐treated). GPMP/TCA care plans were recorded in 15.1% overall, rising to 68.4% and 87.7% in the respective diabetes groups. Among those with diabetes prescriptions, dispensing counts were substantially higher in the insulin‐treated group (median 56, IQR 25–111) than in the non‐insulin–treated group (median 14, IQR 3–53). These administrative features contributed an AUC gain of 0.050 in sensitivity analysis S3.

By Wave 5, self‐reported hypertension had developed in 18.8% of men without diabetes, 31.0% of non‐insulin–treated men and 28.2% of insulin‐treated men. Retention declined from 13,519 at baseline to 5174 at Wave 5 (38.3%). Obesity prevalence increased across all groups (e.g., 14.2%–26.5% in the no‐diabetes group), while high cholesterol rose with age in the no‐diabetes group (10.9%–25.0%) and remained consistently elevated in both diabetes groups. These longitudinal patterns informed the trajectory‐enhanced modelling approach. Full baseline and time‐varying characteristics are provided in Tables A1a–A1b of Appendix A.

### 3.2. Model Performance and Predicted Risk Distribution

All four algorithms achieved similar discrimination (AUROC ≈ 0.77). For XGBoost, AUROC was 0.771 (95% CI: 0.756–0.784) and average precision 0.330 (95% CI: 0.304–0.359) (Supporting Figure S1), with operating characteristics shown in Table 2 and Figure S2. Negative predictive value remained high (0.94–0.97) across thresholds, while positive predictive value (PPV) was low (0.15–0.38), reflecting the 9.7% event rate. At the Youden‐optimal threshold (0.079), sensitivity was 0.753 and specificity 0.657.

**TABLE 2:** Incremental value of feature sets for 10‐year incident hypertension.

Model	Features (*n*)	AUROC (LR)	AUROC (XGBoost)
Age only	1	0.680 (0.664–0.695)	0.672 (0.657–0.688)
Age + BMI	2	0.700 (0.685–0.716)	0.692 (0.677–0.708)
Age + BMI + BMI category	3	0.700 (0.684–0.715)	0.693 (0.678–0.709)
Age + BMI + administrative	6	0.767 (0.752–0.782)	0.764 (0.749–0.778)
Full model	25	0.769 (0.754–0.782)	0.771 (0.756–0.784)

*Note:* Out‐of‐fold AUROC. The administrative variables comprise the GP management plan and team care arrangement status and diabetes indicators. The six‐variable model recovers almost all of the discrimination of the full model; the increment beyond age and BMI is administrative rather than survey‐based.

Accuracy must be interpreted relative to the no‐information rate (0.903); at clinically useful thresholds, it fell below this benchmark (e.g., 0.519 at threshold 0.05), and the F1 score peaked at ∼0.387. Accordingly, discrimination and the sensitivity–specificity–NPV profile are more informative for this low‐prevalence task.

**FIGURE 1:**
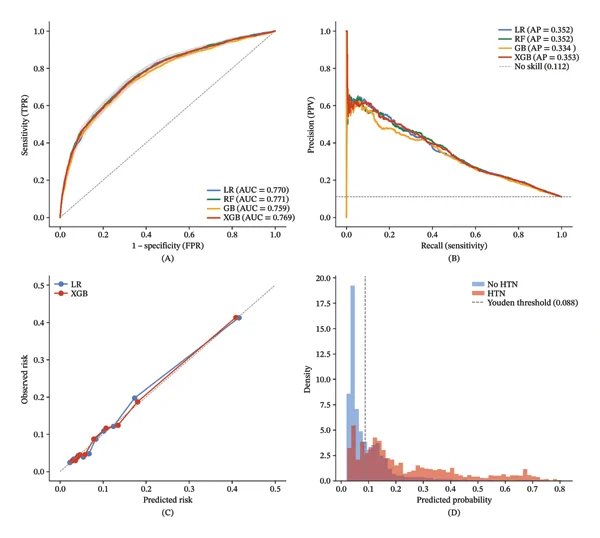
Discrimination and calibration of machine learning models for incident hypertension prediction. (A) ROC curves at a 10‐year horizon. (B) Precision‐recall curves. (C) Calibration plot for 10‐year prediction. (D) Overlapping density histograms of predicted risk probabilities for men who did and did not develop hypertension within.

Calibration curves (Figure 1C) showed good agreement between predicted and observed risk for both logistic regression and XGBoost. Risk deciles demonstrated coherent stratification: the lowest decile comprised younger, leaner men (mean age 17.7 years, BMI 16.4 kg/m^2^) with 1.6% hypertension, while the highest comprised older men (mean age 44.2 years, BMI 26.8 kg/m^2^) with 37.2% hypertension and 30.0% diabetes prevalence (Table A2; Figure A2). The convergence of all models to AUC ≈ 0.77 indicates that performance was constrained by feature information rather than model choice; the small XGBoost advantage (ΔAUC 0.006) does not justify added complexity in this setting [16, 31]. We do not generalise this to other data settings, where more expressive models may confer advantages.

Figure 1D displays overlapping density histograms of predicted risk probabilities for men who did and did not develop hypertension. The event distribution (red) is right‐shifted relative to the non‐event distribution (blue), with a long right tail extending above 0.5. The non‐event distribution is concentrated below 0.15. This confirms that the model successfully shifts predicted risk upward for future cases, though perfect separation is not achieved—consistent with the fundamental uncertainty in 10‐year cardiovascular prediction at AUC = 0.774.

### 3.3. Classification Threshold Performance

The relative best model at a 10‐year prediction was the XGBoost, and we studied five clinically relevant classification thresholds to understand its performance propagation. At a threshold of 0.05 (high sensitivity, low specificity), the model would flag 7219 of 13,519 men (53.4%) as screen‐positive, capturing 85.1% of future hypertension cases but with a PPV of only 15.4%. This threshold is appropriate for initial population‐level screening where the cost of missing a case exceeds the cost of further evaluation. At a threshold of 0.20 (lower sensitivity, higher specificity), 1415 men (10.5%) would be flagged, capturing 39.4% of future cases with a PPV of 36.5% and specificity of 92.6% (Table 3). This threshold is more suitable for targeted intervention programmes where resources are constrained.

**TABLE 3:** Operating characteristics of the primary model (XGBoost, 10‐year incident hypertension), with 95% confidence intervals.

Threshold	Sens.	Spec.	PPV	NPV	F1	Acc.	Pred. +
0.050	0.855 (0.836–0.873)	0.483 (0.475–0.492)	0.151 (0.142–0.159)	0.969 (0.964–0.973)	0.256 (0.244–0.268)	0.519 (0.511–0.528)	7429
0.079^†^	0.753 (0.729–0.776)	0.657 (0.648–0.665)	0.190 (0.180–0.202)	0.961 (0.957–0.965)	0.304 (0.289–0.319)	0.666 (0.658–0.674)	5177
0.100	0.662 (0.637–0.687)	0.738 (0.731–0.746)	0.213 (0.201–0.227)	0.953 (0.949–0.957)	0.323 (0.307–0.340)	0.731 (0.723–0.739)	4062
0.150	0.481 (0.453–0.507)	0.883 (0.877–0.888)	0.306 (0.285–0.326)	0.941 (0.936–0.945)	0.374 (0.353–0.395)	0.844 (0.838–0.850)	2056
0.200	0.395 (0.367–0.422)	0.931 (0.926–0.935)	0.380 (0.353–0.404)	0.935 (0.930–0.939)	0.387 (0.362–0.410)	0.879 (0.873–0.884)	1361

*Note:* Point estimate (95% CI) from 2000 bootstrap replicates of the out‐of‐fold predictions. Overall AUROC 0.771 (95% CI: 0.756–0.784); average precision 0.330 (95% CI: 0.304–0.359); no information rate 0.903. ‘Pred. +’ is the number of men flagged positive at each threshold.

^†^Youden optimal threshold.

The Youden‐optimal threshold (maximising sensitivity + specificity − 1) was 0.079, yielding 72.4% sensitivity and 69.3% specificity, with 4700 men classified as high risk. The negative predictive value exceeded 93% at all thresholds, reflecting the moderate base rate (9.7%) (Table 3). These operating characteristics should be interpreted in the context of the intended use: population‐level risk stratification for preventive intervention, not diagnostic classification.

### 3.4. Feature Importance and Nonlinear Effects

**FIGURE 2:**
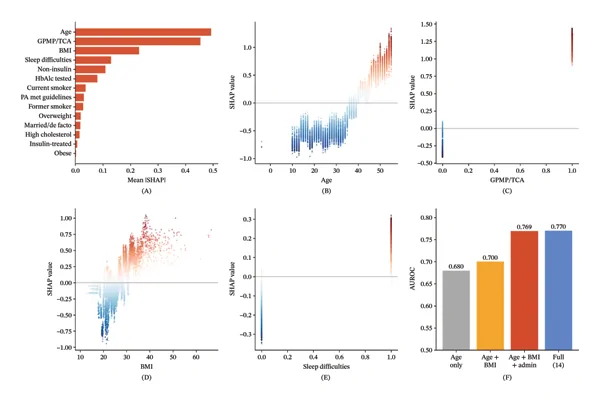
SHAP interpretability analysis of the XGBoost model for 10‐year incident hypertension. (A) Global feature importance ranked by mean absolute SHAP value (top 15 features). (B–E) SHAP dependence plots for the four most important features. (F) Incremental value of data.

SHAP analysis identified a steep importance gradient among the 25 baseline features (Figure 2A; Table A3). The top three features—age (mean |SHAP| = 0.452), GPMP/TCA status (0.336) and BMI (0.188)—collectively dominated model explanatory power. These were followed by GPMP/TCA claims count (0.145), diabetes prescription count (0.073) and alcohol frequency (0.065). Three PBS/MBS‐linked variables appeared among the top six features.

SHAP dependence plots (Figure 2B–E) revealed clinically meaningful nonlinear effects. Age showed accelerating risk above approximately 40 years, consistent with vascular ageing [36]. BMI demonstrated a threshold effect above approximately 30 kg/m^2^, aligning with clinical obesity definitions and known nonlinear adiposity–hypertension relationships [37]. GPMP/TCA claims showed a clear dose–response relationship. Noninsulin diabetes showed a binary positive contribution.

Table A3 extends Figure 2A by providing the full ranking of all 25 baseline features by mean absolute SHAP value, including the 10 features below the display threshold of the main text figure. For each feature, the table reports the mean |SHAP|, the number of participants for whom the feature had a positive SHAP contribution (pushing predicted risk upward) versus a negative contribution and the predominant direction of effect. The complete SHAP analysis for all 25 features (Figure A1) revealed additional patterns: Alcohol frequency showed a J‐shaped relationship consistent with established alcohol–blood pressure associations; physical activity showed a modest negative SHAP shift when guidelines were met. Features ranked 15–25 had mean |SHAP| values below 0.013.

### 3.5. Incremental Value of Administrative Data

To localise the predictive signal, we compared the full model against parsimonious baselines (Table 2, Supporting Figure S3). Age alone achieved an AUROC of 0.680 (logistic regression); adding BMI raised this to 0.700. The substantial increment came from administrative variables: a six‐variable model (age, BMI, BMI category, GP management plan/team care arrangement status and diabetes indicators) achieved an AUROC of 0.767–0.764, recovering approximately 99.5% of the discrimination of the full 25‐feature model. Consistently, restricting it to survey‐only features reduced the XGBoost AUROC from 0.771 to 0.703 (an administrative increment of +0.068).

**FIGURE 3:**
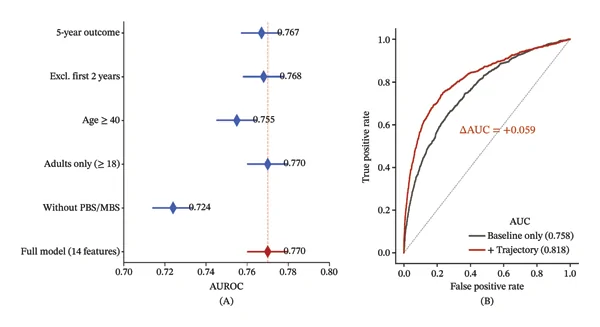
Model robustness and trajectory enhancement. (A) XGBoost AUC‐ROC across six sensitivity conditions. (B) ROC curves comparing baseline‐only versus trajectory‐enhanced XGBoost (*n* = 7188).

Specifically, removing PBS/MBS‐linked features reduced XGBoost AUC from 0.774 to 0.724 (ΔAUC = −0.050; Figure 3A), the largest decrement across all sensitivity conditions. This 5.0‐percentage‐point reduction confirms that pharmaceutical dispensing and healthcare utilisation data provide meaningful, nonredundant predictive information beyond survey features alone. The magnitude is comparable to adding a clinical biomarker to a standard risk model [38].

The differential contribution of PBS/MBS features becomes more apparent across metabolic subgroups. In the no‐diabetes subgroup (99.2% of cohort), PBS/MBS features are less informative—most individuals have zero values. However, in the diabetes subgroup (*n* = 459; 47.3% event rate), diabetes prescription count and GPMP/TCA claims contribute strongly to risk stratification, proxying for unmeasured disease severity [15, 39]. This suggests PBS/MBS‐based risk stratification is most valuable as a targeted strategy for metabolically at‐risk individuals.

Among 7188 participants with three or more waves (879 events; 12.2%), adding six trajectory features improved XGBoost AUC from 0.758 to 0.818 (ΔAUC = +0.059; Figure 3B)—the single largest performance improvement observed. Average precision increased from 0.341 to 0.489 (43% relative improvement).

### 3.6. Longitudinal Surveillance Benchmark and the Trajectory Leakage

**FIGURE 4:**
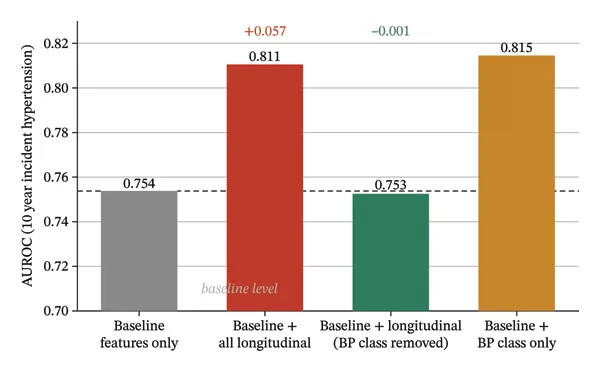
The apparent gain of the longitudinal model is a leakage artefact. Out‐of‐fold AUROC on the subsample of men with at least three waves (*n* = 7188) for the baseline model and for models that add longitudinal features. Removing the single blood pressure trajectory class returns discrimination to the baseline level (dashed line; a change of −0.001), whereas adding that feature alone reproduces almost the entire apparent gain. Because the blood pressure trajectory class incorporates blood pressure status measured during the outcome ascertainment window, it is contemporaneous with rather than antecedent to the outcome.

On the longitudinal subsample (men with at least three waves; *n* = 7,188, 879 events, 12.2%), adding all longitudinal features to the baseline model increased AUROC from 0.754 to 0.811. This gain was, however, entirely attributable to the blood pressure trajectory class: removing it returned discrimination to 0.753 (a change of −0.001), whereas that single feature added to the baseline reproduced almost the whole gain (0.815) (Figure 4). Because this feature incorporates blood pressure status measured during the outcome ascertainment window, the longitudinal model is reported only as a concurrent surveillance benchmark and is not comparable with the baseline prediction models. The genuinely baseline derivable longitudinal features (BMI change and variability, physical activity change and consistency and number of waves) did not improve discrimination.

### 3.7. Subgroup Analysis

**FIGURE 5:**
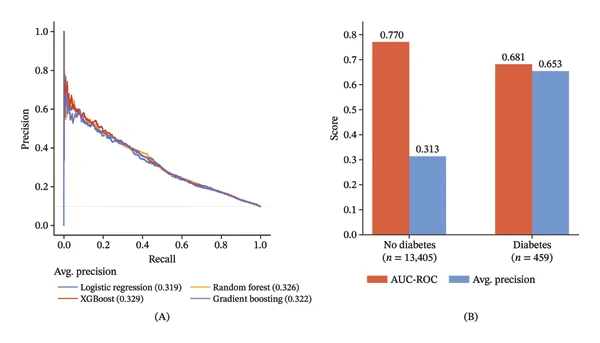
Precision‐recall analysis and subgroup performance. (A) Precision‐recall curves for four models at a 10‐year horizon. (B) Subgroup‐specific XGBoost performance for no‐diabetes and diabetes subgroups.

Subgroup‐specific XGBoost models revealed differential prediction contexts (Figure 5B). In the no‐diabetes subgroup (*n* = 13,405; 9.4% event rate), AUC = 0.770 and average precision = 0.313. In the diabetes subgroup (*n* = 459; 47.3% event rate), AUC was 0.681, but average precision was 0.653—reflecting the different clinical context where the majority develop hypertension.

Precision‐recall analysis (Figure 5A) showed tightly clustered average precision values across all four models, reinforcing the convergence finding. All models achieved average precision approximately 3.3 times the no‐skill baseline (9.7%).

## 4. Discussion

This study examined whether routinely collected survey and administrative data can support clinically meaningful hypertension risk prediction without clinical measurements. The main finding is that an AUC of 0.774 is achievable for 10‐year incident hypertension in Australian men using PBS/MBS‐linked variables alongside survey data—approaching the Framingham benchmark and substantially exceeding prior survey‐only models [9–11]. The performance profile—high negative predictive value but low PPV—indicates suitability for population‐level risk stratification rather than individual diagnosis. At lower thresholds, the model can rule out low‐risk individuals using routine data, reserving clinical assessment for higher‐risk groups; at higher thresholds, it enables targeted prevention with reduced sensitivity. Given persistently low PPV, positive predictions should be interpreted as elevated population risk rather than individual certainty. Beyond overall performance, the findings highlight key insights on the limited incremental value of model complexity, the underutilised potential of administrative data for population surveillance and the role of longitudinal cohorts as platforms for predictive modelling rather than solely descriptive epidemiology.

### 4.1. Model Convergence: Plausible Feature Quality Over Algorithm Selection

Perhaps the most consequential finding for the clinical prediction community is the convergence of all four models—logistic regression, random forest, XGBoost and gradient boosting—to virtually identical discrimination and calibration. This convergence is not an isolated observation; it fits a consistent pattern emerging across the clinical prediction literature. Christodoulou et al. [30], in their landmark review of 71 head‐to‐head comparisons, found that ML methods offered no performance benefit over logistic regression when studies were conducted at low risk of bias. Chowdhury et al. [40] reached a similar conclusion specifically for hypertension risk models, and Liu et al. [41] reported the same for cardiovascular disease prediction from electronic health records more broadly. Most recently, Hwang et al. [42], using large Korean and Japanese national cohorts, reported that XGBoost and logistic regression achieved comparable AUCs for 5‐year incident hypertension prediction using routine health checkup data. The explanation is that when the dominant predictive relationships are approximately log‐linear—as shown by the known associations between age–hypertension and BMI–hypertension—the additional model flexibility of tree‐based ensembles has little leverage and may instead capture noise, especially in situations where moderate class imbalances are present [16, 31, 43]. Incident hypertension was uncommon (9.7% at 10 years). Rather than resampling, we retained the natural class distribution and evaluated performance across a range of decision thresholds. Accuracy is interpreted against the no‐information rate—the accuracy obtained by always predicting the majority (normotensive) class, equal to 0.903—and probability calibration was assessed using the Brier score.

These findings gain additional weight from their replication across two independent analyses of the same cohort. In an exploratory analysis using a self‐reported hypertension outcome and fewer predictors, logistic regression consistently matched or outperformed random forest variants and a stacked ensemble (see complete analysis in Appendix B), the latter demonstrating conclusively that the random forest contributed no unique discriminative signal. Additionally, with an expanded feature set, a PBS‐defined objective outcome and two additional model families (XGBoost and gradient boosting), it reaches the same conclusion at a substantially higher performance level. The consistency of this result across outcome definitions, feature sets and algorithms constitutes strong internal replication. A recent Canadian population‐based study by Chowdhury et al. [40] compared five machine‐learning survival models with conventional Cox regression for predicting incident hypertension and found only marginal performance differences between approaches, with gradient boosting achieving the highest C‐index. This finding reinforces an important practical insight: in prediction tasks, greater gains are often achieved by improving the quality and breadth of input features—such as through administrative data linkage and longitudinal measurements—than by increasing algorithmic sophistication. Although this principle is well recognised in the methodological literature, it has yet to fully permeate clinical prediction practice. While machine learning models offer advantages in more complex settings [19, 44], the assumption that they inherently outperform regression remains widespread, despite strong evidence that predictive performance is primarily driven by data richness, outcome definition and longitudinal depth rather than algorithm choice.

### 4.2. The Prediction Ceiling: What Nonclinical Features Can Achieve

The observed performance requires contextualisation against established benchmarks. The Framingham Hypertension Risk Score, which includes measured blood pressure—the strongest predictor in hypertension models [11, 12]—typically achieves AUCs of 0.78–0.81 [9, 10]. An AUC of 0.774 using entirely nonclinical features therefore approaches this benchmark despite the absence of direct blood pressure measurement. It also improves on prior nonclinical models, including U.K. Biobank analyses (AUC 0.70–0.72) and an EHR‐based model (AUC 0.72) [45, 46]. This gain over earlier work in the same cohort (AUC 0.690) likely reflects the use of an objective PBS‐defined outcome, a richer feature set and improved comorbidity capture.

The narrowing of the AUC gap—from ∼0.08 in survey‐only models to ∼0.01–0.04—highlights the contribution of linked administrative data (Appendix B). PBS and MBS features appear to compensate partly for the absence of measured blood pressure by capturing downstream manifestations of cardiometabolic risk, including medication use, glycaemic monitoring and chronic disease management. This aligns with the concept that administrative data provide a ‘shadow’ of the clinical phenotype through patterns of healthcare utilisation [15]. The remaining performance gap likely reflects the irreducible value of direct blood pressure measurement, which cannot be fully inferred from proxy variables et al. [40]. Taken together, these findings suggest that meaningful risk stratification is achievable without clinical measurements, and at a substantially higher level than previously demonstrated.

### 4.3. Administrative Data: Meaningful Incremental Value

The 0.050 AUC gain from PBS/MBS features reflects the biological and clinical information embedded in healthcare utilisation patterns. GPMP/TCA status and claims frequency capture engagement with structured chronic disease management, indicating established multimorbidity. Diabetes prescription counts proxy treatment intensity and disease severity beyond a binary diagnosis [15, 39], while HbA1c testing frequency reflects monitoring intensity. Together, these variables form an administrative profile of metabolic burden not captured by survey data alone. The magnitude of this improvement is comparable to gains seen when adding clinical biomarkers to standard cardiovascular risk models [38], suggesting administrative data can approximate the predictive value of laboratory measures. Extending prior work on the research utility of PBS/MBS data [14], these findings demonstrate clear value for risk prediction. From a policy perspective, such data represent a scalable population health resource, enabling risk stratification without additional data collection burden.

### 4.4. Surveillance Features: The Prognostic Value of Longitudinal Data

The trajectory‐enhanced model yielded the largest performance gain in this study, exceeding both the PBS/MBS increment and inter‐model differences. This highlights a core principle: cardiovascular risk is dynamic. Individuals with identical BMI at a single timepoint may differ substantially in risk depending on prior trajectories. Features such as BMI change and variability capture metabolic instability, while physical activity consistency reflects sustained behaviour—dimensions not captured cross‐sectionally. The stronger performance relative to earlier trajectory work suggests that predictive gains scale with the richness of engineered longitudinal features, particularly the inclusion of blood pressure trajectory class.

These findings indicate that longitudinal surveys are not merely descriptive but can meaningfully enhance risk prediction, addressing a gap noted in the epidemiological literature [47]. However, inclusion of blood pressure trajectory incorporates intermediate outcome information; thus, the AUC of 0.818 reflects performance achievable within an integrated longitudinal surveillance system rather than a purely baseline model. Even excluding this feature, trajectory variables provide dynamic risk information unavailable from single‐timepoint data, underscoring the added value of longitudinal measurement for prediction.

### 4.5. Hyperparameter Sensitivity

A key methodological finding was the sensitivity of gradient‐boosted models to class weighting. Setting XGBoost’s scale_pos_weight to the class imbalance ratio reduced AUC by 0.042—exceeding the largest performance difference between model families. This pattern, observed across multiple models and analyses, suggests broad relevance for clinical prediction tasks with moderate event rates (5%–15%).

The likely mechanism is distortion of probability rankings: upweighting positives improves sensitivity at the expense of specificity, reducing overall discrimination. While class weighting may be beneficial for very rare outcomes (< 1%), these results indicate it should be treated as a tunable parameter rather than a default adjustment.

Early stopping was equally important. Without it, the 2000‐estimator XGBoost model overfit substantially, with optimal performance achieved at ∼180 trees. This underscores the need for validation‐based regularisation when applying boosting methods in clinical prediction.

### 4.6. SHAP Interpretability and Clinical Translation

SHAP analysis provided both validation of learned relationships and improved interpretability. Dependence plots showed clinically plausible nonlinear effects, including a sharp rise in risk after age 40 years—consistent with vascular ageing mechanisms [36]—and a threshold around BMI 30 kg/m^2^, aligning with obesity‐related hypertension pathways such as RAAS activation and vascular inflammation [37]. Higher GPMP/TCA claim frequency was also associated with increased risk, reflecting greater disease complexity.

These patterns emerged without predefined functional forms, supporting the biological plausibility of model outputs and the value of explainability methods [18, 26]. However, the captured nonlinearities did not materially improve discrimination over logistic regression, as key effects were already represented in standard feature encodings. The main contribution of SHAP was therefore enhanced transparency rather than performance. This interpretability supports clinical translation through threshold‐based decision‐making, enabling models to be adapted for screening, risk stratification or targeted intervention in line with resource and policy constraints.

### 4.7. Australian Policy Implications

These findings address a key gap in Australian hypertension detection. Despite universal healthcare, awareness remains ∼56%, and control rates have stagnated, indicating limits of opportunistic, GP‐based screening [4, 47]. Although national policy prioritises improved detection [5], it lacks mechanisms to systematically identify undiagnosed individuals outside routine care. A model achieving AUC 0.774 using routinely collected PBS/MBS and survey data offers a scalable solution: population‐wide risk stratification without additional clinical encounters. This enables proactive identification and targeted outreach to high‐risk individuals who may otherwise remain undiagnosed.

The male‐specific focus addresses an important gap. Men engage less with primary care and are less likely to have a recent blood pressure measurement [48], increasing their risk of undetected hypertension. A two‐stage approach—administrative data–based triage followed by targeted measurement—could leverage existing Medicare infrastructure without new data collection. Evaluating cost‐effectiveness, including downstream savings from prevented cardiovascular events, is a critical next step for policy translation.

### 4.8. Strengths and Limitations

This study has several methodological strengths. The large, nationally representative cohort with extended follow‐up comprising 142,956 person‐years provides substantial statistical power and supports population‐level generalisability. Defining incident hypertension using PBS‐linked antihypertensive dispensing with precise event dates reduces the misclassification inherent in self‐reported hypertension and represents a meaningful advance over earlier survey‐based analyses. Comprehensive linkage to PBS and MBS administrative records enabled inclusion of features unavailable from surveys alone, while six prespecified sensitivity analyses demonstrated robustness to key modelling and analytical choices. The use of a SHAP‐based interpretability framework addresses requirements for clinical transparency that are increasingly emphasised by regulators and end‐users. Finally, provision of complete replication code in both Python and *R* facilitates cross‐platform verification and supports uptake within the epidemiological research community, where R remains the dominant analytical environment.

Several limitations warrant emphasis. First, hypertension was ascertained from antihypertensive dispensing, which does not capture untreated or lifestyle managed hypertension and may misclassify agents prescribed for other indications. Second, two baseline variables were affected by derivation errors in the analytic extract: country of birth could not be recovered and was excluded, and sleep was ascertainable for only 47% of men; both should be re‐derived from the original survey items before publication. Third, the longitudinal analysis is restricted to men with repeated measures, and the apparent benefit of blood pressure history reflects information contemporaneous with the outcome rather than genuine early prediction. Fourth, the cohort comprises Australian men, and findings may not generalise to women or to other populations.

### 4.9. Future Directions

Key priorities include external validation in independent Australian cohorts with linked data (e.g., the 45 and Up Study) to assess generalisability. The availability of measured blood pressure in later Ten to Men waves will enable benchmarking of the PBS‐defined outcome and support two‐stage prediction strategies combining baseline risk with intermediate measurements.

Methodologically, multiple imputation may improve handling of missing data, while the potential differential value of administrative features across metabolic subgroups warrants validation in larger diabetes cohorts. Evaluation of the cost‐effectiveness of a two‐stage screening approach—administrative triage followed by targeted blood pressure measurement—is also needed. Extending the framework to women will improve generalisability, and incorporating additional administrative indicators (e.g., statin use, mental health medications, specialist referrals and emergency presentations) may further enhance prediction.

## 5. Conclusion

Routinely collected nonclinical data enabled prediction of 10‐year incident hypertension in Australian men with good discrimination (AUC 0.774), exceeding survey‐only models and approaching clinical benchmarks. Similar performance across machine‐learning and regression models indicates that prediction was constrained by feature information rather than algorithm choice. Linked PBS/MBS data provided meaningful incremental value, while longitudinal features yielded the largest gains, highlighting the importance of repeated measurement.

These findings demonstrate that administrative data and longitudinal surveys contain actionable prognostic information and support their integration into population‐scale hypertension risk stratification, enabling proactive, data‐driven screening within the Australian health system.

## Funding

No funding was received for this manuscript. Open access publishing facilitated by Edith Cowan University, as part of the Wiley ‐ Edith Cowan University agreement via the Council of Australasian University Librarians.

## Consent

The authors have nothing to report.

## Conflicts of Interest

The authors declare no conflicts of interest.

## Supporting Information

Additional supporting information can be found online in the Supporting Information section.

## Supporting information


**Supporting Information** The Supporting Information comprises two appendices. Appendix A documents the primary model and its interpretation: a complete predictor dictionary, baseline and across‐wave cohort characteristics by diabetes treatment group, the full SHAP importance ranking with dependence plots for every feature, predicted risk decile profiles with calibration and the predictor correlation structure. Appendix B reports a separate, exploratory analysis of the same cohort using a self‐reported hypertension outcome and a reduced predictor set; across eight classification specifications, a stacked ensemble, a Cox proportional hazards model and a random survival forest, regularised logistic regression matched or exceeded the more flexible learners, giving an internal replication of the algorithm equivalence result under a different outcome definition and feature set. Readers interested in how individual predictors behave are directed to Appendix A; those interested in the robustness of the modelling conclusions across outcomes, features and algorithms are directed to Appendix B.

## Data Availability

The data that support the findings of this study are available on request from the corresponding author. The data are not publicly available due to privacy or ethical restrictions. Noting that Ten to Men data are available to approved researchers through the Australian Data Archive (https://ada.edu.au). PBS‐ and MBS‐linked data are available subject to data custodian approval.

## References

[1] World Health Organization , Global Report on Hypertension: The Race Against a Silent Killer, 2023, WHO.

[2] GBD 2019 Risk Factors Collaborators , Global Burden of 87 Risk Factors in 204 Countries and Territories, 1990–2019, Lancet. (2020) 396, no. 10258, 1223–1249, 10.1016/S0140-6736(20)30752-2. 33069327 PMC7566194

[3] Anto E. O. , Ofori Boadu W. I. , Korsah E. E. et al., Unrecognized Hypertension Among a General Adult Ghanaian Population: An Urban Community-Based Cross-Sectional Study of Prevalence and Putative Risk Factors of Lifestyle and Obesity Indices, PLOS Global Public Health. (2023) 3, no. 5, 10.1371/journal.pgph.0001973. PMC10208459 37224164

[4] Wang X. , Shaw J. E. , Yu J. et al., Prevalence, Awareness, Treatment, and Control Rates of Hypertension in the General Population of Australia: A Systematic Review and Meta-Analysis, Journal of Hypertension. (2025) 43, no. 2, 185–190, 10.1097/hjh.0000000000003854. 39248145

[5] Schutte A. E. , Bennett B. , Chow C. K. et al., National Hypertension Taskforce of Australia. A Roadmap to Achieve 70% Blood Pressure Control in Australia by 2030, Medical Journal of Australia. (2024) 221, no. 3, 126–134, 10.5694/mja2.52373. 38990122

[6] Hird T. R. , Zomer E. , Owen A. J. , Magliano D. J. , Liew D. , and Ademi Z. , Productivity Burden of Hypertension in Australia: A Life Table Modeling Study, Hypertension. (2019) 73, no. 4, 777–784, 10.1161/HYPERTENSIONAHA.118.12606. 30798659

[7] Afrifa-Yamoah E. , Adua E. , Anto E. O. et al., Conceptualised Psycho-Medical Footprint for Health Status Outcomes and the Potential Impacts for Early Detection and Prevention of Chronic Diseases in the Context of 3P Medicine, EPMA Journal. (2023) 14, no. 4, 585–599, 10.1007/s13167-023-00344-2. 38094584 PMC10713508

[8] Anto E. , Frimpong J. , Ofori Boadu W. et al., Cardiometabolic Syndrome Among General Adult Population in Ghana: the Role of Lipid Accumulation Product, Waist Circumference-Triglyceride Index, and Triglyceride-Glucose Index as Surrogate Indicators, Health Science Reports. (2023) 6, no. 7, 10.1002/hsr2.1419. PMC10333904 37441132

[9] Parikh N. I. , Pencina M. J. , Wang T. J. et al., A Risk Score for Predicting Near-Term Incidence of Hypertension: The Framingham Heart Study, Annals of Internal Medicine. (2008) 148, no. 2, 102–110, 10.7326/0003-4819-148-2-200801150-00005. 18195335

[10] Schjerven F. E. , Lindseth F. , and Steinsland I. , Prognostic Risk Models for Incident Hypertension: A PRISMA Systematic Review and Meta-Analysis, PLoS One. (2024) 19, no. 3, 10.1371/journal.pone.0294148. PMC10927109 38466745

[11] Kshirsagar A. V. , Chiu Y. L. , Bomback A. S. et al., Hypertension Risk Score for Middle-Aged and Older Adults, The Journal of Clinical Hypertension. (2010) 12, 800–808. 21029343 10.1111/j.1751-7176.2010.00343.x PMC3683833

[12] Vasan R. S. , Larson M. G. , Leip E. P. , Kannel W. B. , and Levy D. , Assessment of Frequency of Progression to Hypertension in Non-Hypertensive Participants in the Framingham Heart Study: A Cohort Study, Lancet. (2001) 358, no. 9294, 1682–1686, 10.1016/S0140-6736(01)06710-1. 11728544

[13] Muntner P. , Shimbo D. , Carey R. M. et al., Measurement of Blood Pressure in Humans: A Scientific Statement From the American Heart Association, Hypertension. (2019) 73, no. 5, e35–e66, 10.1161/HYP.0000000000000087. 30827125 PMC11409525

[14] Mellish L. , Karanges E. A. , Litchfield M. J. et al., The Australian Pharmaceutical Benefits Scheme Data Collection: A Practical Guide for Researchers, BMC Research Notes. (2015) 8, no. 1, 10.1186/s13104-015-1616-8. PMC4630883 26526064

[15] Islam M. T. , Arafat S. M. , Chowdhury A. et al., Role of the Health Belief Model in the Management of Hypertension: A Systematic Review, Cureus. (2025) 17, no. 10. 10.7759/cureus.94139 PMC12594285 41209884

[16] Goldstein B. A. , Navar A. M. , and Carter R. E. , Moving Beyond Regression Techniques in Cardiovascular Risk Prediction: Applying Machine Learning to Address Analytic Challenges, European Heart Journal. (2017) 38, no. 23, 1805–1814, 10.1093/eurheartj/ehw302. 27436868 PMC5837244

[17] Obermeyer Z. and Emanuel E. J. , Predicting the Future—Big Data, Machine Learning, and Clinical Medicine, New England Journal of Medicine. (2016) 375, no. 13, 1216–1219, 10.1056/NEJMp1606181. 27682033 PMC5070532

[18] Rajkomar A. , Dean J. , and Kohane I. , Machine Learning in Medicine, New England Journal of Medicine. (2019) 380, no. 14, 1347–1358, 10.1056/NEJMra1814259. 30943338

[19] Acheampong E. , Adua E. , Obirikorang C. et al., Predictive Modelling of Metabolic Syndrome in Ghanaian Diabetic Patients: An Ensemble Machine Learning Approach, Journal of Diabetes and Metabolic Disorders. (2024) 23, no. 2, 2233–2249, 10.1007/s40200-024-01491-7. 39610504 PMC11599523

[20] Adua E. , Afrifa-Yamoah E. , and Kolog E. A. , Wang W. , Leveraging Supervised Machine Learning for Determining the Link Between Suboptimal Health Status and the Prognosis of Chronic Diseases, Advances in Predictive, Preventive and Personalised Medicine, 2024, 18, Springer, Cham, 91–113, 10.1007/978-3-031-46891-9_9.

[21] Afrifa-Yamoah E. , Peprah-Yamoah E. , Anto E. O. , Opoku-Yamoah V. , and Adua E. , Protocol for an Integrative Meta-Analysis of the Application of Machine Learning Algorithms in the Prediction of Chronic Disease Risks and Outcomes, Chronic Diseases and Translational Medicine. (2025) 11, no. 3, 1–8, 10.1002/cdt3.70007. 40951737 PMC12426615

[22] Afrifa-Yamoah E. , Adua E. , Peprah-Yamoah E. et al., Pathways to Chronic Disease Detection and Prediction: Mapping the Potential of Machine Learning to the Pathophysiological Processes While Navigating Ethical Challenges, Chronic Diseases and Translational Medicine. (2024) 11, no. 1, 1–21, 10.1002/cdt3.137. 40051825 PMC11880127

[23] Currier D. , Pirkis J. , Carlin J. et al., The Australian Longitudinal Study on Male Health—Methods, BMC Public Health. (2016) 16, no. Suppl 3, 10.1186/s12889-016-3698-1. PMC5103246 28185550

[24] Pirkis J. , Currier D. , Carlin J. et al., Cohort Profile: Ten to Men (the Australian Longitudinal Study on Male Health), International Journal of Epidemiology. (2017) 46, no. 3, 793–794i, 10.1093/ije/dyw055. 27686951

[25] Mosca L. , Barrett-Connor E. , and Wenger N. K. , Sex/Gender Differences in Cardiovascular Disease Prevention, Circulation. (2011) 124, no. 19, 2145–2154, 10.1161/CIRCULATIONAHA.110.968792. 22064958 PMC3362050

[26] Lundberg S. M. , Erion G. , Chen H. et al., From Local Explanations to Global Understanding with Explainable AI for Trees, Nature Machine Intelligence. (2020) 2, no. 1, 56–67, 10.1038/s42256-019-0138-9. PMC7326367 32607472

[27] Pirkis J. , Macdonald J. , and English D. R. , Introducing Ten to Men, the Australian Longitudinal Study on Male Health, BMC Public Health. (2016) 16, no. Suppl 3, 1–4, 10.1186/s12889-016-3697-2. 28185546 PMC5103238

[28] Spittal M. J. , Carlin J. , Currier D. et al., The Australian Longitudinal Study on Male Health Sampling Design and Survey Weighting: Implications for Analysis and Interpretation of Clustered Data, BMC Public Health. (2016) 16, no. Suppl 3, 10.1186/s12889-016-3699-0. PMC5103251 28185562

[29] Collins G. S. , Reitsma J. B. , Altman D. G. , and Moons K. G. M. , Transparent Reporting of a Multivariable Prediction Model for Individual Prognosis or Diagnosis (TRIPOD): The TRIPOD Statement, BMJ. (2015) 350, no. jan07 4, 10.1136/bmj.g7594. 25569120

[30] Christodoulou E. , Ma J. , Collins G. S. , Steyerberg E. W. , Verbakel J. Y. , and Van Calster B. , A Systematic Review Shows No Performance Benefit of Machine Learning over Logistic Regression for Clinical Prediction Models, Journal of Clinical Epidemiology. (2019) 110, 12–22, 10.1016/j.jclinepi.2019.02.004. 30763612

[31] Van Calster B. , McLernon D. J. , van Smeden M. , Wynants L. , and Steyerberg E. , Topic Group ‘Evaluating Diagnostic Tests and Prediction Models’ of the STRATOS Initiative, Calibration: the Achilles Heel of Predictive Analytics. BMC Medicine. (2019) 17, no. 1. 10.1186/s12916-019-1466-7 PMC6912996 31842878

[32] Breiman L. , Random Forests, Machine Learning. (2001) 45, no. 1, 5–32, 10.1023/A:1010933404324.

[33] Wright M. N. and Ziegler A. , Ranger: A Fast Implementation of Random Forests for High Dimensional Data in C++ and R, Journal of Statistical Software. (2017) 77, no. 1, 1–17, 10.18637/jss.v077.i01.

[34] Steyerberg E. W. , Vickers A. J. , Cook N. R. et al., Assessing the Performance of Prediction Models: A Framework for Traditional and Novel Measures, Epidemiology. (2010) 21, no. 1, 128–138, 10.1097/EDE.0b013e3181c30fb2. 20010215 PMC3575184

[35] Harrell F. E. , Lee K. L. , and Mark D. B. , Multivariable Prognostic Models: Issues in Developing Models, Evaluating Assumptions and Adequacy, and Measuring and Reducing Errors, Statistics in Medicine. (1996) 15, no. 4, 361–387. 8668867 10.1002/(SICI)1097-0258(19960229)15:4<361::AID-SIM168>3.0.CO;2-4

[36] Pinto E. , Blood Pressure and Ageing, Postgraduate Medical Journal. (2007) 83, no. 976, 109–114, 10.1136/pgmj.2006.048371. 17308214 PMC2805932

[37] Hall J. E. , do Carmo J. M. , da Silva A. A. , Wang Z. , and Hall M. E. , Obesity, Kidney Function and Blood Pressure Regulation, Nature Reviews Nephrology. (2019) 15, no. 6, 367–385, 10.1038/s41581-019-0145-4. 31015582 PMC7278043

[38] Pencina M. J. , D’Agostino R. B. , and Steyerberg E. W. , Extensions of Net Reclassification Improvement Calculations, Statistics in Medicine. (2011) 30, no. 1, 11–21, 10.1002/sim.4085. 21204120 PMC3341973

[39] Chowdhury E. K. , Owen A. , Krum H. et al., Barriers to Achieving Blood Pressure Treatment Targets in Elderly Hypertensive Individuals, Journal of Human Hypertension. (2013) 27, no. 9, 545–551, 10.1038/jhh.2013.11. 23448846 PMC3747330

[40] Chowdhury M. Z. I. , Naeem I. , Quan H. et al., Prediction of Hypertension Using Traditional Regression and Machine Learning Models: A Systematic Review and Meta-Analysis, PLoS One. (2022) 17, no. 4, 10.1371/journal.pone.0266334. PMC8989291 35390039

[41] Liu T. , Krentz A. , Lu L. , and Curcin V. , Machine Learning Based Prediction Models for Cardiovascular Disease Risk Using Electronic Health Records Data: Systematic Review and Meta-Analysis, European Heart Journal – Digital Health. (2025) 6, no. 1, 7–22, 10.1093/ehjdh/ztae080. 39846062 PMC11750195

[42] Hwang S. H. , Lee H. , Lee J. H. et al., Machine Learning-Based Prediction for Incident Hypertension Based on Regular Health Checkup Data: Derivation and Validation in 2 Independent Nationwide Cohorts in South Korea and Japan, Journal of Medical Internet Research. (2024) 26. 10.2196/52794 PMC11576616 39499554

[43] Boulesteix A.-L. and Schmid M. , Machine Learning Versus Statistical Modeling, Biometrical Journal. (2014) 56, no. 4, 588–593, 10.1002/bimj.201300226. 24615669

[44] Adua E. , Awuni Kolog E. , Afrifa-Yamoah E. et al., Predictive Model and Feature Importance for Early Detection of Type II Diabetes Mellitus, Translational Medicine Communications. (2021) 6, no. 17, 1–15, 10.1186/s41231-021-00096-z.

[45] Alaa A. M. , Bolton T. , Di Angelantonio E. , Rudd J. H. F. , and van der Schaar M. , Cardiovascular Disease Risk Prediction Using Automated Machine Learning: A Prospective Study of Half a Million Adults in the UK Biobank, PLoS One. (2019) 14, no. 5, 10.1371/journal.pone.0213653. PMC6519796 31091238

[46] Ye C. , Fu T. , Hao S. et al., Prediction of Incident Hypertension Within the Next Year: Prospective Study Using Statewide Electronic Health Records and Machine Learning, Journal of Medical Internet Research. (2018) 20, no. 1, 10.2196/jmir.9268. PMC5811646 29382633

[47] Carnagarin R. , Nolde J. M. , Yang J. et al., Stagnating Rates of Blood Pressure Control in Australia: Insights From Opportunistic Screening of 10 046 Participants of the May Measurement Month Campaigns, Journal of Hypertension. (2023) 41, no. 4, 632–637, 10.1097/HJH.0000000000003379. 36723455

[48] Australian Institute of Health and Welfare , High Blood Pressure (Cat. No. PHE 250), 2019, AIHW.

