# Supplementary material for: Machine Learning Prediction of Incident Hypertension in Australian Men: Integrating Survey, Pharmaceutical and Healthcare Utilisation Data From the Ten to Men Cohort
Source: Int J Hypertens. 2026 Aug 25;2026:3041345. doi: 10.1155/ijhy/3041345 (PMC13507513; doi:10.1155/ijhy/3041345)
Supplement: Supplementary file 1 [file IJHY-2026-3041345-s001.docx]

**Supplementary Materials**

**Machine Learning Prediction of Incident Hypertension in Australian Men: Integrating Survey, Pharmaceutical, and Healthcare Utilisation Data from the Ten to Men Cohort**

# *E Afrifa-Yamoah et al.*

# APPENDIX A

# Table A1. Variable Dictionary

The 25 baseline features were drawn from three sources: 16 from the Wave 1 survey, 3 from PBS dispensing records, and 4 from MBS claims data (with 2 survey variables available at all waves). The 6 trajectory features were derived from longitudinal patterns across multiple waves. Of the 25 baseline features, 23 had less than 2% missingness. Household income was the most frequently missing variable (29.0%), handled by median imputation within each cross-validation fold. All PBS/MBS-linked variables had complete data (0.0% missing), as they were derived from administrative records linked to all participants.

The variable types span binary indicators (15 features), ordinal scales (4 features), and continuous measures (6 features among baseline; 4 among trajectory). ATC codes for diabetes classification are specified: A10A for insulin preparations and A10B for oral hypoglycaemic agents. MBS item numbers for HbA1c testing (66551, 66554) and GPMP/TCA chronic disease management plans (721, 723, 732) are documented. BMI cleaning rules (values below 10 or above 70 kg/m² treated as implausible) are noted. This table enables full reproducibility of the feature engineering pipeline.

**Table A1.** Complete predictor variable dictionary for 25 baseline and 6 trajectory features used in machine learning models for incident hypertension prediction. Variables are ordered by SHAP importance rank (baseline features) or listed separately (trajectory features). For each variable, the table reports the exact coding scheme, data source, distributional properties, missingness rate, and mean absolute SHAP value from the XGBoost model trained on the full cohort (N = 13,519).

| **Rank** | **Variable** | **Label** | **Source** | **Type** | **Coding** | **Distribution** | **Miss. (%)** | **Mean \|SHAP\|** |
| --- | --- | --- | --- | --- | --- | --- | --- | --- |
| 1 | age_c | Age | Survey (W1) | Baseline | Continuous (years) | Mean 33.3 (SD 12.8) | 0 | 0.4519 |
| 2 | gpmp_yes | GPMP/TCA recorded | MBS | Baseline | Binary (0/1) | 0=11481 (84.9%) | 0 | 0.336 |
| 3 | bmi_c | BMI | Survey (all) | Baseline | Continuous (kg/m²) | Mean 22.3 (SD 11.2) | 0 | 0.1882 |
| 4 | n_gpmp_claims | GPMP/TCA claims (n) | MBS | Baseline | Count | Mean 0.7 (SD 2.5) | 0 | 0.1448 |
| 5 | sleep_yes | Sleep issues | Survey (W1) | Baseline | Binary (0/1) | 0=7153 (52.9%) | 0 | 0.0928 |
| 6 | n_diab_scripts | Diabetes Rx (n) | PBS | Baseline | Count | Mean 1.7 (SD 15.1) | 0 | 0.0734 |
| 7 | alcohol_ord | Alcohol frequency | Survey (W1) | Baseline | Ordinal (0–4) | 5 levels | 1.7 | 0.0646 |
| 8 | income_ord | Household income | Survey (W1) | Baseline | Ordinal (1–5) | 5 levels | 29 | 0.0533 |
| 9 | rural_inner | Inner regional | Survey (W1) | Baseline | Binary (0/1) | 0=10561 (78.1%) | 0 | 0.0437 |
| 10 | pa_met | Physical activity | Survey (all) | Baseline | Binary (0/1) | 1=7867 (58.2%) | 0 | 0.0401 |
| 11 | hba1c_yes | HbA1c tested | MBS | Baseline | Binary (0/1) | 0=12997 (96.1%) | 0 | 0.0317 |
| 12 | grp_noninsulin | Non-insulin DM | PBS | Baseline | Binary (0/1) | 0=13174 (97.4%) | 0 | 0.031 |
| 13 | edu_uni | University education | Survey (W1) | Baseline | Binary (0/1) | 0=10690 (79.1%) | 0 | 0.0217 |
| 14 | rural_outer | Outer regional/remote | Survey (W1) | Baseline | Binary (0/1) | 0=10934 (80.9%) | 0 | 0.0193 |
| 15 | smoker_current | Current smoker | Survey (W1) | Baseline | Binary (0/1) | 0=11263 (83.3%) | 0 | 0.0127 |
| 16 | married | Married/de facto | Survey (W1) | Baseline | Binary (0/1) | 1=7411 (54.8%) | 0 | 0.0098 |
| 17 | smoker_former | Former smoker | Survey (W1) | Baseline | Binary (0/1) | 0=10959 (81.1%) | 0 | 0.0093 |
| 18 | bmi_cat_ord | BMI category | Survey (W1) | Baseline | Ordinal (0–3) | 4 levels | 0 | 0.0072 |
| 19 | n_hba1c | HbA1c tests (n) | MBS | Baseline | Count | Mean 0.2 (SD 1.4) | 0 | 0.0045 |
| 20 | edu_cert | Certificate/diploma | Survey (W1) | Baseline | Binary (0/1) | 0=8701 (64.4%) | 0 | 0.0021 |
| 21 | high_chol | High cholesterol | Survey (W1) | Baseline | Binary (0/1) | 0=12259 (90.7%) | 0 | 0.0017 |
| 22 | cob_overseas | Born overseas | Survey (W1) | Baseline | Binary (0/1) | Invariant | 0 | 0 |
| 23 | stroke_yes | Stroke history | Survey (W1) | Baseline | Binary (0/1) | 0=13476 (99.7%) | 0 | 0 |
| 24 | heart_cond | Heart condition | Survey (W1) | Baseline | Binary (0/1) | 0=13447 (99.5%) | 0 | 0 |
| 25 | grp_insulin | Insulin-treated DM | PBS | Baseline | Binary (0/1) | 0=13405 (99.2%) | 0 | 0 |
| – | n_waves | Waves observed | Derived | Trajectory | Count (1–5) | 5 levels | 0 | – |
| – | bmi_change | BMI change | Derived | Trajectory | Continuous | Mean 1.0 (SD 3.2) | 6.2 | – |
| – | bmi_std | BMI variability | Derived | Trajectory | Continuous | Mean 1.1 (SD 1.6) | 0 | – |
| – | pa_prop_met | PA consistency | Derived | Trajectory | Continuous (0–1) | Mean 0.6 (SD 0.4) | 0 | – |
| – | pa_change | PA change | Derived | Trajectory | Categorical (-1/0/1) | 3 levels | 0 | – |
| – | bp_traj_class | BP trajectory class | Derived | Trajectory | Categorical (0/1/3) | 0=12087 (89.4%) | 0 | – |

*Note.* DM = diabetes mellitus; Rx = prescriptions; GPMP = GP Management Plan; TCA = Team Care Arrangement; PBS = Pharmaceutical Benefits Scheme; MBS = Medicare Benefits Schedule; PA = physical activity; W1 = Wave 1. Trajectory features available only for subsample with ≥3 waves (n = 7,188). SHAP ranks based on mean absolute SHAP value from XGBoost model trained on full cohort.

**Table A1a.** Baseline-only demographic, socioeconomic, and PBS/MBS-linked characteristics by diabetes treatment group (Wave 1)

| **Baseline-only characteristic** | **No diabetes** | **Non-insulin treated** | **Insulin-treated** | **Overall** |
| --- | --- | --- | --- | --- |
| **Education** |  |  |  |  |
| University degree | 2716 (34.1) | 93 (36.9) | 20 (27.8) | 2829 (34.2) |
| Certificate/diploma | 4628 (58.2) | 142 (56.3) | 48 (66.7) | 4818 (58.2) |
| Year 12 or equivalent | 610 (7.7) | 17 (6.7) | 4 (5.6) | 631 (7.6) |
| **Household income** |  |  |  |  |
| <$30,000 | 597 (6.5) | 25 (8.1) | 7 (7.9) | 629 (6.5) |
| $30,000–$59,999 | 1664 (18.1) | 80 (26.0) | 22 (24.7) | 1766 (18.4) |
| ≥$60,000 | 6947 (75.4) | 203 (65.9) | 60 (67.4) | 7210 (75.1) |
| Missing | 3852 (29.5) | 37 (10.7) | 25 (21.9) | 3914 (29.0) |
| **Remoteness** |  |  |  |  |
| Major Cities | 7708 (59.0) | 204 (59.1) | 62 (54.4) | 7974 (59.0) |
| Inner Regional | 2853 (21.8) | 73 (21.2) | 32 (28.1) | 2958 (21.9) |
| Outer Regional/Remote | 2497 (19.1) | 68 (19.7) | 20 (17.5) | 2585 (19.1) |
| **Marital status** |  |  |  |  |
| Married/de facto | 7091 (65.4) | 265 (78.9) | 55 (51.4) | 7411 (65.7) |
| Never married | 3151 (29.1) | 49 (14.6) | 39 (36.4) | 3239 (28.7) |
| **PBS/MBS-linked** |  |  |  |  |
| HbA1c tested (MBS) | 203 (1.6) | 216 (62.6) | 103 (90.4) | 522 (3.9) |
| GPMP/TCA recorded (MBS) | 1702 (13.0) | 236 (68.4) | 100 (87.7) | 2038 (15.1) |
| Any diabetes prescriptions | 0 (0.0) | 345 (100.0) | 114 (100.0) | 459 (3.4) |
| Diabetes scripts, median (IQR)† | 0 (0–0) | 14 (3–53) | 56 (25–111) | 20 (4–69) |
| GPMP/TCA claims, median (IQR)† | 3 (2–5) | 6 (3–10) | 7 (4–13) | 3 (2–6) |
| HbA1c tests, median (IQR)† | 1 (1–1) | 3 (2–6) | 9 (4–14) | 2 (1–5) |

*†Among those with at least one record. PBS = Pharmaceutical Benefits Scheme; MBS = Medicare Benefits Schedule; GPMP = GP Management Plan; TCA = Team Care Arrangement; HbA1c = glycated haemoglobin. Household income: 29.0% missing overall.*

**Table A1b.** Time-varying characteristics of the incident hypertension cohort across waves by baseline diabetes treatment group (n = 13,519)

|  | **Wave 1** | | | **Wave 2** | | | **Wave 3** | | | **Wave 4** | | | **Wave 5** | | | |
| --- | --- | --- | --- | --- | --- | --- | --- | --- | --- | --- | --- | --- | --- | --- | --- | --- |
| **Characteristic** | ***No DM*** | ***Non-ins*** | ***Insulin*** | ***No DM*** | ***Non-ins*** | ***Insulin*** | ***No DM*** | ***Non-ins*** | ***Insulin*** | ***No DM*** | ***Non-ins*** | ***Insulin*** | ***No DM*** | ***Non-ins*** | ***Insulin*** |  |
| N | 13060 | 345 | 114 | 9594 | 293 | 86 | 6304 | 199 | 59 | 5589 | 181 | 58 | 4980 | 154 | 40 |  |
| Age, mean (SD) | 33.0 (12.8) | 42.2 (9.5) | 38.2 (12.6) | 35.6 (13.1) | 45.0 (9.3) | 41.3 (11.7) | 41.2 (13.0) | 50.6 (8.8) | 46.7 (11.7) | 43.6 (12.8) | 52.5 (8.7) | 49.0 (11.9) | 45.4 (12.7) | 54.8 (8.3) | 48.1 (11.7) |  |
| BMI, mean (SD) | 25.8 (5.0) | 31.1 (5.7) | 28.2 (6.3) | 26.3 (5.1) | 31.4 (5.7) | 28.7 (6.8) | 27.0 (4.8) | 31.4 (6.0) | 29.2 (6.7) | 27.3 (5.0) | 31.8 (6.1) | 29.2 (5.7) | 27.6 (5.0) | 31.5 (5.5) | 29.7 (7.1) |  |
| **BMI category** |  |  |  |  |  |  |  |  |  |  |  |  |  |  |  |  |
| Normal weight | 4555 (34.9) | 36 (10.4) | 31 (27.2) | 3252 (33.9) | 23 (7.8) | 21 (24.4) | 1755 (27.8) | 13 (6.5) | 13 (22.0) | 1467 (26.2) | 10 (5.5) | 14 (24.1) | 1228 (24.7) | 7 (4.5) | 10 (25.0) |  |
| Overweight | 4486 (34.3) | 109 (31.6) | 32 (28.1) | 3389 (35.3) | 97 (33.1) | 21 (24.4) | 2719 (43.1) | 57 (28.6) | 19 (32.2) | 2446 (43.8) | 49 (27.1) | 17 (29.3) | 2153 (43.2) | 53 (34.4) | 11 (27.5) |  |
| Obese | 1858 (14.2) | 162 (47.0) | 33 (28.9) | 1649 (17.2) | 147 (50.2) | 32 (37.2) | 1417 (22.5) | 117 (58.8) | 22 (37.3) | 1369 (24.5) | 111 (61.3) | 25 (43.1) | 1322 (26.5) | 88 (57.1) | 18 (45.0) |  |
| Met PA guidelines | 7627 (58.4) | 184 (53.3) | 56 (49.1) | 5825 (60.7) | 161 (54.9) | 49 (57.0) | 4174 (68.2) | 115 (57.8) | 31 (53.4) | 3418 (61.2) | 87 (48.1) | 27 (46.6) | 3519 (70.7) | 90 (58.4) | 20 (50.0) |  |
| Self-reported high BP | 0 (0.0) | 0 (0.0) | 0 (0.0) | 426 (5.1) | 34 (12.0) | 7 (8.6) | 517 (8.4) | 48 (24.7) | 10 (17.5) | 565 (15.7) | 60 (36.4) | 12 (21.4) | 637 (18.8) | 45 (31.0) | 11 (28.2) |  |
| **Smoking status** |  |  |  |  |  |  |  |  |  |  |  |  |  |  |  |  |
| Non-smoker | 3357 (42.2) | 104 (38.2) | 34 (40.0) | 2684 (44.2) | 95 (41.5) | 28 (43.8) | 2462 (39.8) | 74 (38.1) | 25 (42.4) | 1502 (56.3) | 45 (52.9) | 13 (43.3) | 1364 (60.8) | 35 (58.3) | 8 (50.0) |  |
| Former smoker | 2442 (30.7) | 95 (34.9) | 23 (27.1) | 1993 (32.9) | 85 (37.1) | 22 (34.4) | 3027 (48.9) | 91 (46.9) | 25 (42.4) | 542 (20.3) | 19 (22.4) | 6 (20.0) | 391 (17.4) | 12 (20.0) | 4 (25.0) |  |
| Current smoker | 2155 (27.1) | 73 (26.8) | 28 (32.9) | 1389 (22.9) | 49 (21.4) | 14 (21.9) | 702 (11.3) | 29 (14.9) | 9 (15.3) | 626 (23.4) | 21 (24.7) | 11 (36.7) | 489 (21.8) | 13 (21.7) | 4 (25.0) |  |
| High cholesterol | 1168 (10.9) | 70 (21.2) | 22 (22.4) | 998 (12.0) | 66 (23.2) | 17 (21.0) | 901 (14.7) | 60 (30.8) | 18 (31.0) | 798 (22.2) | 61 (37.4) | 13 (23.2) | 848 (25.0) | 53 (36.6) | 12 (30.0) |  |
| Sleep issues | 6114 (100.0) | 183 (100.0) | 69 (100.0) | 4304 (100.0) | 140 (100.0) | 46 (100.0) | 3099 (100.0) | 99 (100.0) | 29 (100.0) | 2656 (100.0) | 91 (100.0) | 22 (100.0) | 2333 (100.0) | 70 (100.0) | 18 (100.0) |  |
| Stroke | 40 (0.4) | 3 (0.9) | 0 (0.0) | 43 (0.5) | 2 (0.7) | 1 (1.2) | 38 (0.6) | 0 (0.0) | 2 (3.5) | 39 (1.1) | 1 (0.6) | 1 (1.8) | 47 (1.4) | 2 (1.4) | 0 (0.0) |  |
| Heart condition | 66 (0.6) | 6 (1.8) | 0 (0.0) | 57 (0.7) | 5 (1.8) | 1 (1.2) | — | — | — | — | — | — | — | — | — |  |
| **Alcohol use** |  |  |  |  |  |  |  |  |  |  |  |  |  |  |  |  |
| Never | 2678 (20.9) | 72 (21.2) | 17 (14.9) | 1553 (16.4) | 53 (18.7) | 11 (13.3) | — | — | — | — | — | — | — | — | — |  |
| 4+ times/week | 2582 (20.1) | 63 (18.6) | 28 (24.6) | 2076 (21.9) | 50 (17.7) | 18 (21.7) | — | — | — | — | — | — | — | — | — |  |

*Values are n (%) unless otherwise indicated. — = not available at this wave. Percentages from non-missing values. Diabetes group defined by PBS dispensing (ATC A10A/A10B) and fixed at baseline.*

# Table A2. Predicted Risk Decile Profile

The model stratified the cohort into clinically meaningful risk groups. The lowest risk decile (decile 1) comprised young men (mean age 17.7 years, mean BMI 16.4 kg/m²) with a predicted risk of 2.5% and observed event rate of 1.6%, no diabetes representation, and 71.7% meeting physical activity guidelines. The highest risk decile (decile 10) comprised older men (mean age 44.2 years, mean BMI 26.8 kg/m²) with a predicted risk of 36.5%, observed event rate of 37.2%, 30.0% diabetes prevalence, and only 51.4% meeting physical activity guidelines.

The monotonic increase in observed event rates across deciles (1.6% to 37.2%) confirms good calibration and clinical face validity. The progressive enrichment of diabetes in higher deciles—from 0.0% in deciles 1–5 to 30.0% in decile 10—demonstrates that the model appropriately recognises diabetes as a major risk factor without being exclusively driven by it (70% of the top decile are non-diabetic). Sleep issues prevalence showed a non-monotonic pattern, peaking in the middle deciles, consistent with its role as a modifier rather than a dominant predictor. High cholesterol prevalence increased steadily from 0.6% in decile 1 to 22.2% in decile 10. These profiles support the model’s clinical coherence and could inform risk-stratified screening strategies.

**Table A2.** Clinical characterisation of predicted risk deciles from the XGBoost model for 10-year incident hypertension (N = 13,519). Each decile contains approximately 1,352 men, stratified by out-of-fold predicted probabilities. Mean predicted risk, observed event rate, and clinical profile variables (age, BMI, physical activity, high cholesterol, diabetes prevalence, and sleep issues) are reported per decile to assess calibration and clinical face validity.

| **Decile** | **N** | **Events** | **Pred. risk** | **Obs. rate** | **Mean age** | **Mean BMI** | **PA met (%)** | **Hi chol (%)** | **DM (%)** | **Sleep (%)** |
| --- | --- | --- | --- | --- | --- | --- | --- | --- | --- | --- |
| 1 | 1352 | 21 | 0.025 | 0.016 | 17.7 | 16.4 | 71.7 | 0.6 | 0.0 | 2.0 |
| 2 | 1354 | 41 | 0.030 | 0.031 | 22.2 | 16.9 | 63.9 | 1.0 | 0.0 | 30.1 |
| 3 | 1350 | 49 | 0.035 | 0.036 | 23.3 | 18.5 | 59.9 | 2.5 | 0.0 | 58.7 |
| 4 | 1352 | 49 | 0.034 | 0.036 | 27.2 | 20.8 | 52.4 | 2.6 | 0.0 | 65.5 |
| 5 | 1352 | 57 | 0.048 | 0.042 | 32.3 | 23.4 | 57.0 | 5.5 | 0.0 | 58.4 |
| 6 | 1351 | 96 | 0.064 | 0.071 | 37.2 | 25.6 | 58.0 | 10.4 | 0.2 | 47.4 |
| 7 | 1352 | 134 | 0.088 | 0.099 | 41.5 | 25.0 | 58.6 | 13.3 | 0.4 | 44.4 |
| 8 | 1352 | 145 | 0.115 | 0.107 | 43.7 | 23.8 | 55.6 | 16.6 | 0.7 | 48.9 |
| 9 | 1352 | 215 | 0.159 | 0.159 | 43.6 | 25.9 | 53.4 | 18.6 | 2.7 | 58.3 |
| 10 | 1352 | 503 | 0.365 | 0.372 | 44.2 | 26.8 | 51.4 | 22.2 | 30.0 | 57.2 |

*Note.* Pred. risk = mean predicted probability; Obs. rate = observed event rate; PA met = met physical activity guidelines; Hi chol = high cholesterol; DM = diabetes (any type). Deciles based on XGBoost out-of-fold predicted probabilities.

# Table A3. Complete SHAP Feature Ranking

The ranking reveals a steep importance gradient. The top three features—age (mean |SHAP| = 0.452), GPMP/TCA status (0.336), and BMI (0.188)—collectively accounted for the majority of model explanatory power. Features ranked 11–25 had mean |SHAP| values below 0.032, indicating minimal individual contribution. Notably, four features had zero SHAP contributions across all participants: country of birth (entirely invariant in this cohort), stroke history, heart condition, and insulin-treated diabetes. The zero contribution of insulin-treated diabetes reflects its very low prevalence (114 of 13,519; 0.8%), meaning the model had insufficient positive instances to learn a reliable signal—in contrast to non-insulin treated diabetes (345 participants, mean |SHAP| = 0.031).

The directional analysis reveals that most features predominantly decrease predicted risk when present (labelled ‘Risk ↓’). This is because the majority of the cohort (90.3%) did not develop hypertension, so the model’s baseline prediction is weighted toward non-events. Features like GPMP/TCA status show asymmetric directionality: of 13,519 participants, only 2,038 (15.1%) had GPMP/TCA recorded, but their SHAP contributions were consistently positive (risk-increasing), while the 11,481 without GPMP/TCA received negative contributions. Alcohol frequency and HbA1c test count were the only features where positive SHAP contributions outnumbered negative ones among the full cohort, suggesting these features act primarily as risk-elevating indicators when present at higher levels.

**Table A3.** Complete SHAP (SHapley Additive exPlanations) feature ranking for all 25 baseline predictors from the XGBoost model for 10-year incident hypertension (N = 13,519). Features are ranked by mean absolute SHAP value. For each feature, the number of participants with positive SHAP contributions (pushing predicted risk upward) and negative SHAP contributions (pushing predicted risk downward) are reported, along with the predominant direction of effect across the cohort.

| **Rank** | **Feature** | **Label** | **Mean \|SHAP\|** | **+SHAP (n)** | **–SHAP (n)** | **Direction** |
| --- | --- | --- | --- | --- | --- | --- |
| 1 | age_c | Age | 0.4519 | 4,972 | 8,547 | Risk ↓ |
| 2 | gpmp_yes | GPMP/TCA recorded | 0.336 | 2,038 | 11,481 | Risk ↓ |
| 3 | bmi_c | BMI | 0.1882 | 4,216 | 9,303 | Risk ↓ |
| 4 | n_gpmp_claims | GPMP/TCA claims (n) | 0.1448 | 2,002 | 11,517 | Risk ↓ |
| 5 | sleep_yes | Sleep issues | 0.0928 | 6,366 | 7,153 | Risk ↓ |
| 6 | n_diab_scripts | Diabetes Rx (n) | 0.0734 | 500 | 13,019 | Risk ↓ |
| 7 | alcohol_ord | Alcohol frequency | 0.0646 | 7,152 | 6,367 | Risk ↑ |
| 8 | income_ord | Household income | 0.0533 | 4,626 | 8,893 | Risk ↓ |
| 9 | rural_inner | Inner regional | 0.0437 | 2,958 | 10,561 | Risk ↓ |
| 10 | pa_met | Physical activity | 0.0401 | 5,657 | 7,862 | Risk ↓ |
| 11 | hba1c_yes | HbA1c tested (MBS) | 0.0317 | 662 | 12,857 | Risk ↓ |
| 12 | grp_noninsulin | Non-insulin DM | 0.031 | 345 | 13,174 | Risk ↓ |
| 13 | edu_uni | University education | 0.0217 | 2,849 | 10,670 | Risk ↓ |
| 14 | rural_outer | Outer regional/remote | 0.0193 | 2,616 | 10,903 | Risk ↓ |
| 15 | smoker_current | Current smoker | 0.0127 | 2,255 | 11,264 | Risk ↓ |
| 16 | married | Married/de facto | 0.0098 | 6,108 | 7,411 | Risk ↓ |
| 17 | smoker_former | Former smoker | 0.0093 | 2,732 | 10,787 | Risk ↓ |
| 18 | bmi_cat_ord | BMI category | 0.0072 | 3,676 | 9,843 | Risk ↓ |
| 19 | n_hba1c | HbA1c tests (n) | 0.0045 | 10,937 | 2,582 | Risk ↓ |
| 20 | edu_cert | Certificate/diploma | 0.0021 | 7,308 | 6,211 | Risk ↑ |
| 21 | high_chol | High cholesterol | 0.0017 | 9,445 | 4,074 | Risk ↑ |
| 22 | cob_overseas | Born overseas | 0 | 0 | 0 | – |
| 23 | stroke_yes | Stroke history | 0 | 0 | 0 | – |
| 24 | heart_cond | Heart condition | 0 | 0 | 0 | – |
| 25 | grp_insulin | Insulin-treated DM | 0 | 0 | 0 | – |

*Note.* +SHAP (n) = number of participants with positive SHAP contribution; –SHAP (n) = negative contribution. Direction = predominant direction of association for majority of participants. DM = diabetes mellitus; Rx = prescriptions.

# Figure A1. SHAP Dependence Plots for All 25 Baseline Features

**Link to main text:** Figure A1 extends Figure 2B–E by presenting SHAP dependence plots for all 25 baseline features, arranged in a 5 × 5 grid ordered by importance rank (left to right, top to bottom). This figure allows readers to inspect the functional form of every feature’s effect, complementing the top-4 plots shown in the main manuscript.

**Figure A1.** *SHAP dependence plots for all 25 baseline features, arranged in a 5 × 5 grid ordered by SHAP importance rank. Each panel shows feature value (x-axis) versus marginal SHAP contribution to predicted hypertension risk (y-axis). Points above the horizontal zero line indicate positive contributions to predicted risk.*

The plots reveal several patterns beyond the top four features discussed in the main text. GPMP/TCA claims count (rank 4) shows a clear dose–response relationship: SHAP values increase progressively with the number of claims, consistent with the interpretation that higher healthcare utilisation intensity proxies for greater disease burden. Sleep issues (rank 5) show a clean binary split, with presence associated with a positive SHAP shift of approximately +0.08. Alcohol frequency (rank 7) shows a J-shaped relationship: abstainers and very low-frequency drinkers have near-zero SHAP values, while higher-frequency consumption (3–4 on the ordinal scale) is associated with modestly increased predicted risk—consistent with established alcohol–blood pressure associations. Physical activity (rank 10) shows a modest negative SHAP shift when guidelines are met.

Features ranked 15–25 show SHAP values tightly concentrated near zero with minimal dispersion, confirming their limited individual predictive contribution. Country of birth shows a single point at zero SHAP, reflecting its invariance after preprocessing. These low-importance features were retained in the model as their inclusion did not harm performance (confirmed by sensitivity analyses) and they contribute to marginal improvements through interactions captured by the tree ensemble.

# Figure A2. Risk Decile Calibration and Clinical Profile

**Figure A2.** Risk decile calibration and clinical profile. (A) Predicted versus observed event rates across risk deciles as side-by-side bars. (B) Mean age (bars) and diabetes prevalence (purple line, right axis) across deciles.

Panel A shows close correspondence between predicted and observed rates across all 10 deciles—from 2.5% predicted / 1.6% observed in decile 1 to 36.5% predicted / 37.2% observed in decile 10—providing decile-level calibration evidence. The slight underprediction in decile 1 and slight overprediction in deciles 7–8 are within the range expected from sampling variability (n ≈ 1,352 per decile). Panel B reveals a striking inflection in diabetes prevalence: absent from deciles 1–4, appearing marginally in deciles 5–7 (0.2–0.7%), accumulating in decile 9 (2.7%), and reaching 30.0% in decile 10. This non-linear enrichment demonstrates that diabetes contributes to risk prediction in a threshold-like fashion, elevating individuals into the highest-risk stratum.

# Figure A3. Feature Correlation Matrix

**Link to main text:** Figure A3 addresses a methodological concern inherent to any multivariable prediction model: the degree of collinearity among predictor variables. This figure complements the Methods section (Section 2.4, Predictor Variables) by showing the pairwise Pearson correlation structure across all 25 baseline features.

**Figure A3.** Pairwise Pearson correlation matrix for all 25 baseline features. Colour intensity indicates correlation magnitude; blue indicates positive correlation, red indicates negative correlation.

The strongest correlation was between age and marital status (r = 0.60), reflecting the demographic association between ageing and partnership formation. Age was also moderately correlated with alcohol frequency (r = 0.45), BMI category (r = 0.35), and certificate/diploma education (r = 0.30). Among PBS/MBS-linked features, GPMP/TCA status and GPMP/TCA claim count were correlated (r = 0.68), as one is the binary indicator and the other the continuous count of the same administrative process. GPMP/TCA claims correlated with diabetes prescription count (r = 0.41), reflecting co-management of chronic conditions.

Importantly, no correlations approached the level that would compromise model stability for tree-based methods (which are inherently robust to collinearity). SHAP analysis confirmed that each feature contributed independently to predictions—age, BMI, and marital status all retained non-zero SHAP values. The logistic regression model, which is more sensitive to collinearity, achieved AUC within 0.006 of XGBoost, further indicating that collinearity did not meaningfully distort performance.

**APPENDIX B**

# Supplementary Table B1. Predictor Variable Summary

This table provides the complete specification of all predictor variables used in the analysis. Variables marked ‘Both’ were included in both the non-redundant 16-predictor set (used by logistic regression and tuned random forest) and the full 19-predictor set (used by the default random forest). Variables marked ‘Full only’ were included only in the 19-predictor set. Trajectory features were available only for the subsample contributing data at three or more waves (N = 6,174).

| **Variable** | **Type** | **Source** | **Missing (%)** | **Feature set** | **Notes** |
| --- | --- | --- | --- | --- | --- |
| Age | Continuous | Survey | 0.0 | Both | Years at Wave 1 |
| BMI (continuous) | Continuous | Survey | 0.0 | Both | kg/m² |
| BMI (category) | 4-level | Survey | 0.0 | Full only | UW/Normal/OW/Obese |
| Age category | 5-level | Survey | 0.0 | Full only | 10–19 to 50+ |
| Diabetes treatment | 3-level | Survey/PBS | 0.0 | Both | None/Non-insulin/Insulin |
| Smoking status | 3-level+Miss | Survey | 39.7 | Both | Non/Former/Current |
| Physical activity | Binary | Survey | 0.0 | Both | Met/Did not meet |
| Alcohol use | 5-level+Miss | Survey | 1.7 | Both | Never to 4+/week |
| Rurality | 3-level | Survey | 0.0 | Both | Major cities/Inner/Outer |
| Marital status | 3-level+Miss | Survey | 17.7 | Both | Married/Never/Other |
| Heart condition | Binary+Miss | Survey | 18.6 | Both | Yes/No |
| Stroke history | Binary+Miss | Survey | 18.7 | Full only | Yes/No |
| High cholesterol | Binary+Miss | Survey | 18.6 | Both | Yes/No |
| GPMP/TCA status | Binary | MBS | 0.0 | Both | Any claim recorded |
| N diabetes scripts | Count | PBS | 43.6† | Both | Imputed 0 if missing |
| N HbA1c tests | Count | MBS | 41.2† | Both | Imputed 0 if missing |
| N GPMP/TCA claims | Count | MBS | 41.2† | Both | Imputed 0 if missing |
| HbA1c tested | Binary | MBS | 0.0 | Both | Yes/No |
| BMI slope‡ | Continuous | Derived | NA | Trajectory | β of BMI on wave |
| BMI variability‡ | Continuous | Derived | NA | Trajectory | SD across waves |
| PA stability‡ | Proportion | Derived | NA | Trajectory | Prop. waves met PA |

** Sleep: 53.8% not reported; treated as ‘No’. +Miss = missing coded as explicit factor level. † PBS/MBS counts: % without linked records; imputed to zero. ‡ Trajectory features: calculated from repeated measures across ≥3 waves.*

Feature set curation rationale: Preliminary analysis identified that including both continuous and categorical versions of the same construct diluted random forest variable importance by splitting discriminative signal across correlated representations. The non-redundant set removed categorical BMI and age category while retaining the continuous forms. The full set was retained for the default random forest to quantify the redundancy effect (AUC reduction of 0.014; main text Section 3.4).

Figure B1. Exploratory analysis of hypertension incidence and predictor distributions. (A) Incidence by age and diabetes group; (B) by BMI and diabetes group; (C) BMI density by outcome; (D) age distribution; (E) PBS/MBS feature distributions; (F) by smoking and physical activity.

## Classification model comparison

Table B1 presents the complete eight-model comparison from 5-fold cross-validation. Logistic regression with non-redundant features achieved the highest AUC (0.690, SD 0.027), closely followed by the stacked LR+RF ensemble (0.689, SD 0.020) and LR with interaction terms (0.689, SD 0.025). The weighted LR achieved nominally the highest AUC (0.691) but substantially worse calibration (Brier 0.223 vs. 0.064), reflecting the distortion introduced by inverse-probability weighting. Among RF variants, the tuned model (0.678, SD 0.010) represented a meaningful improvement over the default (0.664, SD 0.012). The down-sampled RF (0.669) fell between default and tuned. The class-balanced RF performed worst at 0.645. Sensitivity at 80% specificity—a clinically relevant threshold for population screening—ranged from 0.332 (class-balanced RF) to 0.424 (LR and stacked ensemble). ROC curves are shown in Figure 2. The comprehensive diagnostic panel including fold-by-fold stability and calibration plots is available as Supplementary Figure S1.

***Table B1.*** *Classification model comparison (5-fold stratified cross-validation). Sens@80% = sensitivity at 80% specificity. IPW = inverse probability weighting.*

| **Model** | **AUC** | **SD** | **Brier** | **Sens@80%** | **Approach** |
| --- | --- | --- | --- | --- | --- |
| Weighted LR | 0.691 | 0.027 | 0.223 | 0.417 | IPW |
| LR (non-redundant) | 0.690 | 0.027 | 0.064 | 0.424 | Regression |
| Stacked (LR+RF) | 0.689 | 0.020 | 0.065 | 0.424 | Ensemble |
| LR + interactions | 0.689 | 0.025 | 0.064 | 0.417 | Regression |
| RF (tuned) | 0.678 | 0.010 | 0.064 | 0.382 | ML |
| RF (downsampled) | 0.669 | 0.015 | 0.108 | 0.373 | ML |
| RF (default) | 0.664 | 0.012 | 0.065 | 0.377 | ML |
| RF (class-balanced) | 0.645 | 0.013 | 0.109 | 0.332 | ML |

*Figure 2. ROC curves for logistic regression and random forest models (5-fold cross-validated). The comprehensive diagnostic panel including fold-by-fold stability and calibration plots is available as Supplementary Figure S1.*

# Supplementary Figure B2. Comprehensive Diagnostic Panel

**Supplementary Figure B2.** *Comprehensive diagnostic panel for the eight-model classification comparison. (A) AUC comparison with fold-level uncertainty, showing all models ranked by mean cross-validated AUC. (B) ROC curve overlay for the top five models. (C) Fold-by-fold AUC stability. (D) Calibration by predicted risk decile.*

**Interpretation.** This panel extends the summary in main text Table B1 by providing visual detail on the sources of performance differences. The ROC overlay (Panel B) confirms that logistic regression variants trace nearly identical curves, while random forest variants diverge primarily in the mid-specificity range (60–80%)—precisely where clinical screening trade-offs are most consequential. The calibration plots (Panel D) reveal that the class-balanced and down-sampled RF approaches produce substantially mis-calibrated risk estimates (Brier 0.108–0.109 vs. 0.064 for LR), undermining their utility for risk estimation even where discrimination is adequate. The fold-by-fold stability plot (Panel C) illustrates a bias-variance trade-off: RF models achieve lower variance at the cost of lower mean performance, consistent with their tendency to average toward the majority class under class imbalance.

## **3.4 Random Forest diagnostic analysis**

Four factors contributed to the default RF’s inferior performance. First, the 1:13 class imbalance caused Gini-based splits to favour the majority class, producing predicted probabilities compressed toward the low base rate (7.1%) and reducing discrimination in the clinically relevant mid-probability range. Second, redundant features—particularly the inclusion of both continuous and categorical encodings of BMI and age—diluted split importance across correlated variables; removing these redundancies and using the non-redundant 16-predictor set improved AUC from 0.664 to 0.678. Third, high-cardinality categorical variables (alcohol with 6 levels including ‘Missing’, marital status with 4 levels) created a large partition search space. With sparse events in many factor-level combinations, the RF overfitted to noise rather than signal. Fourth, ranger’s categorical encoding, which evaluates all possible binary partitions of factor levels at each split, is computationally expensive and prone to overfitting with many-levelled factors, whereas logistic regression handles these parsimoniously via dummy coding.

Counterintuitively, class-weight balancing (inverse-prevalence weighting) worsened RF performance from 0.664 to 0.645. By inflating each positive instance’s weight by ~13-fold, the balanced RF amplified idiosyncratic patterns in individual event cases, effectively trading underfitting of the minority class for overfitting to noise. Down-sampling to a 3:1 ratio (0.669) was more effective than full re-weighting but still underperformed tuned hyperparameters without resampling (0.678). The stacked ensemble’s parity with standalone LR (0.689 vs. 0.690) is the most diagnostic finding: the RF base learner contributed no discriminative information beyond what LR already captured, confirming the absence of exploitable non-linear structure in this feature space.

## **3.5 Prediction ceiling analysis**

The ceiling analysis (Figure B3) addressed the fundamental question of why overall model performance was moderate. Univariate feature AUCs revealed that the strongest individual predictor was age (AUC 0.667), followed by continuous BMI (0.646). All other features showed markedly weaker univariate discrimination. PBS/MBS features were barely above chance: HbA1c test count (0.508), diabetes script count (0.506), and GPMP/TCA claims (0.503).

Forward feature selection demonstrated rapid diminishing returns. Age alone contributed AUC of approximately 0.66; adding BMI raised this to approximately 0.68; each subsequent feature added less than 0.01 AUC, with the curve plateauing near 0.70 regardless of additional features. This plateau matches the performance of the best models (LR: 0.690) and approaches the UK Biobank survey-only benchmark (~0.71) [15], confirming that the models extract nearly all available signal from the feature set. The approximately 0.08 AUC gap between the best model and the Framingham clinical benchmark (~0.78) [7] quantifies the information value of measured blood pressure and laboratory tests for hypertension prediction. This gap represents an inherent limitation of non-clinical feature sets rather than a modelling deficiency.

Figure B3. Ceiling analysis. (A) Incremental AUC by greedy forward feature selection (red dashed line = Framingham clinical benchmark at ~0.78); (B) observed vs. predicted event rates by decile for best model.

## **3.8 Subgroup analysis**

Separate random forest models fitted within diabetes treatment strata revealed differential predictor importance (Figure B4). In the no-diabetes group (N = 11,794; 823 events), BMI and age dominated importance, followed by lifestyle factors (alcohol use, smoking, rurality). In the non-insulin treated diabetes subgroup (N = 156; 20 events), the pattern shifted substantially: diabetes script count became the second most important predictor after BMI, surpassing age, and HbA1c test frequency also ranked higher than in the general population model. This suggests that while PBS/MBS features contribute minimally to population-level prediction, they become clinically relevant precisely in the metabolic subgroups that stand to benefit most from targeted screening. Given the small subgroup size and limited number of events, these findings should be considered hypothesis-generating rather than definitive. The convergence of all model families at AUC 0.68–0.69 (Supplementary Figure S4) reinforces the ceiling analysis finding that performance is bounded by feature informativeness.

*Figure B4. Subgroup variable importance: no diabetes (N = 11,794; 823 events) vs. non-insulin treated diabetes (N = 156; 20 events). The overall model comparison across all approaches is available as Supplementary Figure S4.*

# Supplementary Figure B5. Random Survival Forest Variable Importance

**Supplementary Figure B5.** *Random survival forest variable importance based on impurity splitting. The model was fitted using ranger with 300 trees, minimum terminal node size of 20, and 16 predictors.*

**Interpretation.** The RSF importance ranking broadly parallels the classification RF importance (main text Figure 4), with age and BMI dominant. Differences include relatively greater RSF weight on alcohol use frequency and marital status—both of which have time-varying associations with hypertension that the survival framework can partially capture. GPMP/TCA claims rank similarly in both approaches, reinforcing the consistency of this administrative feature’s modest but reproducible predictive contribution. The RSF’s overall lower discrimination (C-index 0.585 vs. classification RF AUC 0.678) reflects the coarse time approximation rather than any deficiency of the random forest algorithm in the survival setting.

The Cox proportional hazards model achieved a concordance index of 0.682, comparable to the best classification models and confirming that the time-to-event framing did not substantially improve discrimination. In the adjusted Cox model (Figure 5), continuous BMI was the strongest predictor. Current smoking (HR ~1.7), physical inactivity, sleep problems, and GPMP/TCA status were independently associated with incident hypertension. PBS features (diabetes script count, HbA1c tests) showed modest non-significant hazard ratios after full adjustment, consistent with their near-chance univariate AUCs—their value lies in subgroup-specific prediction rather than population-level discrimination.

*Figure B6. Cox proportional hazards forest plot with adjusted hazard ratios and 95% confidence intervals. PBS/MBS administrative features are highlighted.*

The random survival forest achieved a lower C-index of 0.585, likely reflecting the coarse time approximation from wave participation. Kaplan-Meier curves stratified by diabetes group and RSF variable importance are available as Supplementary Figures S2. The curves demonstrate clear separation between diabetes groups, with the non-insulin treated group showing the steepest decline in hypertension-free survival. The no-diabetes group (98.2% of the cohort) shows the slowest rate of hypertension development. The insulin-treated curve should be interpreted cautiously given its very small sample (N = 60; 5 events). Widening confidence bands beyond 6 years reflect attrition (retention 36.9% at Wave 5). The coarse time approximation from wave participation introduces imprecision that likely attenuated survival model discrimination, partly explaining the random survival forest’s low C-index (0.585) relative to classification models (AUC up to 0.690).

*Figure B7.* *Kaplan-Meier hypertension-free survival curves stratified by diabetes treatment group (no diabetes, non-insulin treated, insulin-treated), with number at risk table and log-rank test for group differences. Follow-up time approximated from wave participation.*

# Supplementary Figure B8. Overall Model Discrimination Comparison

**Supplementary Figure B8.** Discrimination across all primary modelling approaches: eight classification models (AUC from 5-fold CV), Cox proportional hazards (Harrell’s C-index), and random survival forest (out-of-bag C-index). Error bars represent standard deviations or bootstrap confidence intervals*.*

**Interpretation.** This summary figure provides a unified visual comparison across all model families. Logistic regression variants and the stacked ensemble cluster tightly at AUC 0.689–0.691, confirming the robustness of LR across specifications. Random forest variants show a clear ordering reflecting progressive benefit of feature curation and hyperparameter optimisation: class-balanced (0.645) < default (0.664) < down-sampled (0.669) < tuned (0.678). The Cox model’s concordance (0.682) is competitive with the best classification models; the RSF (0.585) underperforms all other approaches due to the coarse time approximation.

The convergence of LR, stacked ensemble, and Cox model at approximately 0.68–0.69 provides strong evidence that this represents the practical discrimination ceiling for the available non-clinical feature set. No model—regardless of algorithmic family—exceeded AUC 0.691, reinforcing the ceiling analysis conclusion (main text Figure 3) that performance is bounded by feature informativeness at approximately 0.70.
